# CRISPR Technologies in Chinese Hamster Ovary Cell Line Engineering

**DOI:** 10.3390/ijms24098144

**Published:** 2023-05-02

**Authors:** Katja Glinšek, Krištof Bozovičar, Tomaž Bratkovič

**Affiliations:** Faculty of Pharmacy, University of Ljubljana, Aškerčeva 7, 1000 Ljubljana, Slovenia

**Keywords:** Chinese hamster ovary cells, cell line development, CRISPR-Cas, genome editing, glycoengineering, augmenting productivity

## Abstract

The Chinese hamster ovary (CHO) cell line is a well-established platform for the production of biopharmaceuticals due to its ability to express complex therapeutic proteins with human-like glycopatterns in high amounts. The advent of CRISPR technology has opened up new avenues for the engineering of CHO cell lines for improved protein production and enhanced product quality. This review summarizes recent advances in the application of CRISPR technology for CHO cell line engineering with a particular focus on glycosylation modulation, productivity enhancement, tackling adventitious agents, elimination of problematic host cell proteins, development of antibiotic-free selection systems, site-specific transgene integration, and CRISPR-mediated gene activation and repression. The review highlights the potential of CRISPR technology in CHO cell line genome editing and epigenetic engineering for the more efficient and cost-effective development of biopharmaceuticals while ensuring the safety and quality of the final product.

## 1. Introduction

Chinese Hamster Ovary (CHO) cells are the most prevalent host cell line for the production of biopharmaceuticals [1,2]. CHO cells were first used to express the tissue plasminogen activator, approved for clinical use in 1987 [3], which was the first recombinant therapeutic protein expressed in mammalian cells. Ever since, CHO cells have remained the preferred mammalian expression system for the large-scale commercial manufacturing of various biopharmaceuticals, such as monoclonal antibodies (mAbs), human growth hormones, cytokines, and clotting factors [1,3,4,5]. Data on biopharma market approvals between 2014 and 2018 reveal that over 80% of newly approved biopharmaceuticals were produced in CHO cells [4] and this number has risen to 89% until 2022 [6], indicating that this trend is to continue. mAbs are dominating the biopharmaceutical approvals, followed by hormones. If the clotting factors and enzymes were in 3rd and 4th place among approved types of biologics between 2014 and 2018 [4], they were replaced by nucleic acid/gene therapy-based products and vaccines in the following 4-year period [6]. The popularity of CHO cells is not surprising since they possess many characteristics beneficial for commercial protein production. First, their ability to grow in suspension instead of adherent cultures makes them suitable for high-volume bioreactors [3]. They were adapted to grow in a chemically defined serum-free medium, which simplifies downstream processes and minimizes potential regulatory obstacles. Another characteristic that makes CHO cells regulatory-friendly is their safety regarding the replication of human pathogenic viruses. One of their key features is the ease of integrating foreign DNA and the stable expression of recombinant proteins with human-like post-translational modifications in sufficient yields [1,2,3]. Tremendous improvements have been made in CHO cell line engineering in the past few decades, and this is especially noticeable by improving cell productivity, stability, product quality, and safety [7]. When the genome sequence of the CHO-K1 cell line became available in 2011 [8], it gave this field a tremendous boost. Together with the advances in proteomics, transcriptomics, epigenomics, metabolomics, and glycomics [5], the elucidation of the CHO cell genome sequence facilitated detailed characterization and major advances in the engineering of this industrial workhorse which we partly cover in this review.

**Figure 1:**
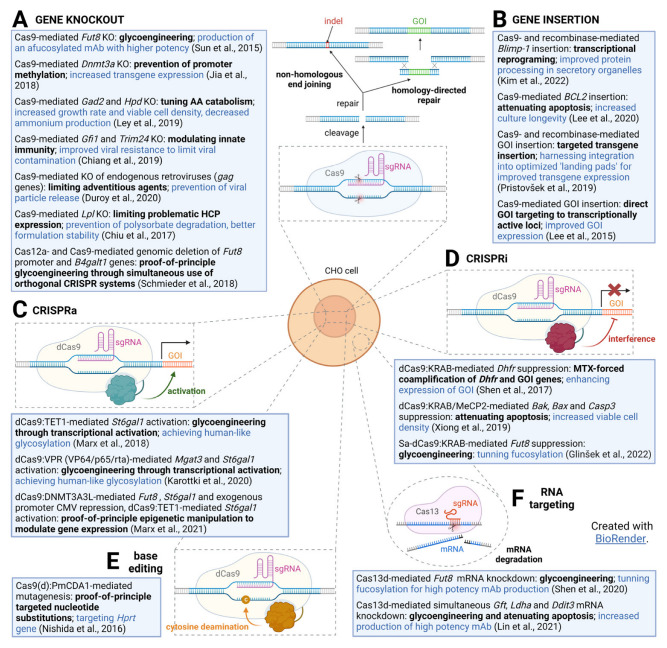
Summary of diverse CRISPR-Cas system applications in CHO cell line. (**A**) Gene knockout [17,18,19,20,21,22,23]; (**B**) targeted gene knockin [24,25,26,27]; (**C**) CRISPR activation [28,29,30]; (**D**) CRISPR interference [31,32,33]; (**E**) DNA base editing [34]; (**F**) targeted mRNA knockdown [35,36].

Genetic tools enabling programmable and sequence-specific genome editing are being widely exploited in all areas of life sciences. The value of these tools arises from their capability to be versatile for engineering cells and organisms with high specificity and efficiency [9,10,11]. Common to all three programmable nucleases (zinc-finger nucleases (ZFNs), transcription activator-like effector nucleases (TALENs), and RNA-guided engineered nucleases (RGENs) derived from the bacterial CRISPR (clustered regularly interspaced short palindromic repeat)-Cas (CRISPR-associated system) is their ability to induce double-strand breaks (DSBs) at specific sites in the genome, thereby triggering repair through endogenous mechanisms (e.g., nonhomologous end joining (NHEJ), microhomology-mediated end joining (MMEJ), and homology-directed repair (HDR) pathways) [9,11]. NHEJ and MMEJ result in gene disruption when the target site is in the gene coding region, while HDR can lead to gene insertion, correction, or point mutagenesis [11]. ZFNs and TALENs are fusions of modular sequence-specific DNA-binding protein domains and nonspecific *Fok*I nuclease, while in the two-component CRISPR-Cas9 system, the innate nuclease activity of Cas9 is directed to the target site by the user-specified short guide RNA (gRNA) [9,12]. Several review papers provide an excellent overview and comparison of all three systems [10,11,12,13,14,15]. All three systems were utilized to engineer CHO cells, but due to the simplicity and relative ease of gRNA design [16], CRISPR prevailed over other genome editing techniques. This review sums up the CRISPR-Cas9 system applications in CHO cell line engineering (Figure 1).

## 2. CRISPR-Cas9 System

The first publications in 2013, demonstrating the capability of the CRISPR-Cas9 system to engineer human cells [37,38,39], triggered a revolution in genome editing that reached laboratories all around the globe [40]. The reason for the widespread and rapid adoption of CRISPR technology lies in its simple RNA-DNA interactions for targeting. In contrast to earlier ZFNs and TALENs, which use protein-DNA interactions, complex and labor-intensive protein design is no longer needed, because RGENs use simple Watson–Crick base-pairing rules in directing engineered RNA to the target DNA [13,40]. The technology originates from CRISPR-Cas systems, an adaptive immune system used by bacteria to protect themselves from invading viruses and plasmids [40]. From several different CRISPR-Cas systems discovered, the type II CRISPR-Cas9 system from *Streptococcus pyogenes* is the most widely adopted due to its simple build [12,41]. It requires only CRISPR-associated protein 9 (Cas9) nuclease, CRISPR RNA (crRNA), and transactivating RNA (tracRNA). The tool was simplified further by fusing crRNA with tracRNA, creating single guide RNA (sgRNA) [41]. In a two-component system, sgRNA guides Cas9 nuclease to the DNA target site, complementary to the 5′ end of sgRNA. Cas9 then cleaves any complementary sequence located next to a short protospacer adjacent motif (PAM), having the sequence NGG in the case of *S. pyogenes* Cas9. Thus, Cas9 nuclease can be guided to any N_20_-NGG pattern sequence by simply designing a sgRNA with the 20-nucleotide target-specific sequence [13,41]. The system has also been repurposed for other applications, such as the modulation of gene expression, chromatin or DNA modification, imaging of specific genomic regions, and RNA cleavage [13,16]. Mutations in both nuclease domains of Cas9 (D10A and H840A) led to the creation of the nuclease inactive Cas9 variant, dubbed «dead Cas9» (dCas9) [42,43]. The dead variant lacks target DNA cleavage activity but retains selective DNA binding ability as specified by sgRNA. When fused to the effector domains, e.g., transcription activators or repressors, and epigenetic modulators, such genetic tools can be used for the manipulation of gene expression or epigenetic regulation [41]. Manipulating this protein has broadened the application of CRISPR-Cas9 technology, and such tools were also used in the engineering of CHO cell lines.

## 3. CRISPR-Cas9-Mediated Gene Deletion in CHO Cells

The CRISPR-Cas9 system has been utilized in CHO cell line engineering to modify numerous endogenous genes, generating production cell lines with desired phenotypes, including increased productivity and growth characteristics, targeted glycosylation profiles, enhanced immunity against viral contamination, and the elimination of problematic host cell proteins. In the following sections, a detailed overview of different cellular pathways disrupted by CRISPR-Cas9 in CHO cell lines is presented (summarized in Table 1).

### 3.1. Glycosylation

Protein glycosylation is a complex post-translational modification of both endogenous and therapeutic proteins [44,45]. Glycosylation plays a significant role in protein folding and stability, biological activity, immune responses, and affects serum half-life, and it is therefore considered a critical quality attribute (CQA) of protein therapeutics [46]. The non-template-driven nature of the glycosylation process makes this post-translational modification difficult to control during the bioprocess, and glycans attached to the proteins are typically heterogeneous. CHO cells produce glycoproteins decorated with N-glycans, similar but not identical to those of human cells [47]. Much effort has been put into the glycoengineering of CHO cells to expand their glycosylation capacity, to achieve either a more homogeneous population of N-glycans or fully humanized N-glycans on therapeutic proteins [48,49].

One of the most comprehensive studies in glycoengineering of CHO cells was conducted by Yang et al. [49]. The authors performed a knock-out (KO) screen of 19 glycosyltransferase genes involved in N-glycan galactosylation, branching, poly-LacNac elongation, terminal sialylation, and fucosylation. Single or a combination of genes were targeted by ZFNs, TALENs, and CRISPR-Cas9. The role of each glycosyltransferase in N-glycosylation was examined on erythropoietin (EPO), a model protein stably expressed by CHO cells. On top of the valuable information about the individual glycosyltransferase’s role in the formation of N-glycans, the study provided evidence that engineering of the N-glycosylation pathway does not affect O-glycosylation capacity or compromise cell growth and productivity [49]. Although the majority of KOs were performed by ZFNs, the generated knowledge facilitated subsequent glycoengineering studies exploiting other genome editing techniques by providing information about the most suitable targets to achieve the desired glycoprofile [48].

Monoclonal antibodies (mAbs) are the predominant product type in the universe of biopharmaceutical approvals and global sales, and the majority represent anti-cancer antibodies [4]. The mode of action of some anti-cancer IgG1 mAbs relies on triggering the Fc-mediated immune effector functions, such as antibody-dependent cellular cytotoxicity (ADCC) [50]. Such antibodies recognize specific tumor-associated antigens expressed on cancer cells. The Fab region binds to the antigen, while the Fc region binds to the Fc receptor FcγRIII on natural killer cells and consequently activates the ADCC response [50]. Fc-FcγRIII interaction is highly affected by the glycan structure present at the N-glycosylation site Asn297 in each of the CH2 domains of the antibody [51]. It is widely known that the removal of core fucose from the Fc region increases the affinity between Fc and FcγRIII and therefore enhances ADCC activity [50,52,53]. In the pre-CRISPR era, different approaches were employed to generate fucose-free antibodies, from traditional homologous recombination [54] to ZFNs [55]. The most common target is alpha-(1,6)-fucosyltransferase (FUT8), a glycoenzyme that catalyzes the transfer of fucose from GDP-fucose to *N*-acetylglucosamine (GlcNAc) [54]. It is the only enzyme mediating the attachment of core fucose to the N-glycans in mammalian cells [56] and therefore an obvious target for fucosylation engineering.

The first report about CRISPR-Cas9-mediated KO of the *Fut8* gene in CHO cells [57] was soon followed by a demonstration of the industrial potential of CRISPR-Cas9 technology to produce afucosylated antibodies [17]. The authors targeted exon 10 encoding for the catalytic site of the FUT8 enzyme in the CHO-K1 host cell line. Because only mutations in both alleles, *Fut8*^−/−^, produced completely afucosylated glycoproteins, an additional lectin-based (*Lens culinaris* agglutinin (LCA)) phenotypic screen was performed to enrich only *Fut8*^−/−^ clones. A comparison of antibodies expressed from the *Fut8*^−/−^ host cell line and wild-type CHO-K1 host confirmed that the new host produced only afucosylated antibodies. Moreover, functional KO of the *Fut8* gene did not impair cell growth, viability, or product quality [17]. Although the data were not shown, the authors discussed that the KO of the gene encoding for GDP-fucose transporter (*Slc35c1*), responsible for transferring GDP-fucose into the Golgi, did not affect fucosylation levels. A year later, a study reporting the opposite was published [58]. Here, three different genome editing techniques, ZFNs, TALEN, and CRISPR-Cas9, were utilized to inactivate the *Slc35c1* gene in anti-Her2 mAb-producing CHO cells [58]. The targeted gene was disrupted with all three approaches, and the following FACS coupled with fucose-specific *Aleuria aurantia* lectin (AAL) enabled the enrichment of mutant cells with the reduced fucose phenotype. Moreover, similar to the *Fut8* KO study [17], the inactivation of the *Slc35c1* gene did not impair cell growth. Taken together, this study suggests that the disruption of the GDP-transporter is another feasible approach to produce fucose-free antibodies [58]. Additionally, the study confirmed the advantage of the FACS enrichment step to swiftly obtain the desired phenotype.

An alternative approach for reducing fucosylation on mAbs was reported by Joubert et al. [59], who expressed a membrane-associated anti-FUT8 intrabody that inhibits FUT8 activity and leads to reduced cell surface fucosylation on CHO cells. Moreover, the coexpression of the intrabody construct and the antibody rituximab led to the generation of clones producing rituximab with varying fucosylation levels, with the maximum fucose reduction rate of around 90%.

Sialic acids, terminal monosaccharides of complex N-glycans, are crucial for the quality and stability of therapeutic proteins [60], and sialylation is thus a popular target for glycoengineering of CHO cells. The presence of sialic acids prevents glycoproteins from binding to the liver asialoglycoprotein receptor that recognizes terminal galactose and mediates protein intake. Terminal sialic acids mask galactose residues and therefore contribute to a longer serum half-life of glycoproteins [61,62]. On human proteins, both α-2,6- and α-2,3-linked terminal sialic acids are present [61]. The beta-galactoside alpha-2,6-sialyltransferase 1 gene (*St6gal1*) is present but silenced in CHO cells [28], characterizing glycoproteins produced by these cell lines with incomplete sialylation including only α-2,3-linked sialic acid. Therapeutic proteins possessing both types of sialic acid linkage are more human-like and considered less immunogenic [61]. Moreover, it was reported that Fc α-2,6-sialylation contributes to the anti-inflammatory activity of intravenous immunoglobulins (IVIG) [63]. Chung et al. [64] have addressed the challenge of producing exclusively α-2,6-sialylated antibodies in CHO cells, which they achieved by combining amino acid substitution of the IgG Fc region with CRISPR-Cas9-mediated KOs of two α-2,3-sialyltransferases, ST3GAL4 and ST3GAL6, and overexpression of ST6GAL1. Amino acid substitution made the Fc region more accessible to glycosyltransferases. Combining these three approaches resulted in more than 77% of almost exclusively α-2,6-sialylated IgGs.

An interesting observation related to the sialylation in CHO cells was reported by Fischer et al. [65]. CHO cells are known to express a scarce amount of *N*-glycolylneuraminic acid (NGNA) compared to other nonhuman mammalian cell lines, such as mouse myeloma NSO and SP2/0 cell lines [66]. This makes the CHO cell line the preferable option for producing therapeutic proteins, since the loss-of-function mutation in cytidine monophosphate (CMP)-N-acetylneuraminic acid hydroxylase (*Cmah*) gene, responsible for converting *N*-acetylneuraminic acid (NANA) to CMP-NGNA, is present in humans [67]. The presence of the *Cmah* gene in CHO cells was confirmed [8], but the reason for its low expression is not known. An observation of unusually high levels of NGNA in one of the mAb-producing CHO cell lines revealed that the loss of the small non-coding RNA (*cgr*-miR-111) was responsible for the upregulation of the *Cmah* gene, resulting in increased NGNA levels [65].

The heterogeneity of CHO N-glycans is especially undesirable when a distinct glycan structure is a prerequisite for a therapeutic protein. α1-antitrypsin (A1AT) and C1 esterase inhibitor (C1INH) are two examples of human plasma proteins that possess homogeneous and distinct glycoprofiles and are used as plasma-derived augmentation therapy to treat α1-antitrypsin deficiency (AATD) or hereditary angioedema (HAE-C1INH), respectively [68]. Both proteins primarily possess two-antennary afucosylated glycans with human-like α-2,6-linked sialic acid [69]. Since augmentation therapy is associated with high costs and the risk of viral infection, the recombinant production of such molecules represents a cost-effective and safe alternative. CRISPR-Cas9 was utilized to engineer CHO cells for the production of A1AT and C1INH with humanized N-glycans [68]. This was achieved by CRISPR-Cas9-mediated KO of 10 genes (*Mgat4A*, *Mgat4B*, *Mgat5*, *St3gal3*, *St3gal4*, *St3gal6*, *B3gnt2*, *Fut8*, *Sppl3,* and *Glul*) involved in glycosylation of CHO cells [49], and overexpression of ST6GAL1. Both A1AT and C1INH expressed in this glycoengineered CHO cell line carry homogeneous glycostructures similar to the human plasma-derived proteins. The study showed that recombinant A1AT produced in CRISPR-Cas9-glycoengineered CHO cells is a great alternative to cost-intensive augmentation therapy [68]. Moreover, an extended half-life of A1AT was demonstrated with KOs of *St3gal4* and *Fut8* and overexpressing ST6GAL1 compared to conventional recombinant A1AT leading to a more native-like glycosylation profile [70].

The same group used a similar CRISPR-Cas9 multiplexed approach to produce agalactosylated monoclonal antibodies. Amman et al. [71] studied the activity of four different beta-1,4-galactosyltransferases (B4GALTs) involved in CHO N-glycosylation in an industrial-relevant CHO-S cell line. Combinatorial KOs using CRISPR-Cas9 of *B4galt1*, *B4galt2*, *B4galt3*, and *B4galt4* revealed important conclusions for CHO glycoengineering, e.g., disruption of *B4galt1*, *B4galt2*, and *B4galt3* led to predominantly agalactosylated secreted proteins (rituximab and EPO) while no contribution to N-glycosylation was detected for *B4galt4*.

Another comprehensive glycoengineering study was published in 2019 by Tian et al. [72]. They investigated glycoengineering options for lysosomal replacement enzymes, the most prevailing therapy for rare lysosomal storage diseases (LSDs), and one of the most complex challenges in the development of biopharmaceuticals [73,74]. Although being essential therapy for LSDs, recombinant lysosomal replacement enzymes are only partially effective in clinical use, mainly due to inefficient delivery to hard-to-reach organs and short circulatory half-life [72]. A thorough screening of glycoengineering options for lysosomal enzymes was performed, generating a large number of CHO cell lines capable of producing these enzymes with glycosylation patterns impacting cellular uptake and circulation. The authors targeted 43 genes involved in N-glycosylation and mannose 6-phosphate processing, individually or in combinations, and showed an improved circulation time and delivery to the heart of glycoengineered alpha-galactosidase capped with α-2,3-linked sialic acid in a Fabry disease mouse model.

While extensive knowledge has been generated on N-glycosylation, a lot less is known about O-glycosylation and its impact on the safety and efficacy of biologics. In 2022, a paper reporting on the O-glycoengineered CHO cell platform was published [75]. While other studies reported targeting genes involved in O-glycosylation in CHO cells [76,77], this was the first study in which the glycoengineered CHO cell line platform was generated to assess the impact of different O-glycan structures on therapeutic proteins [75]. The model protein in the study was etanercept, a dimeric fusion protein with 3 N-glycosylation and 13 O-glycosylation sites. The mentioned platform was generated by CRISPR-Cas9-mediated KOs of different genes involved in O-glycosylation, demonstrating that therapeutic proteins with desired O-glycans can be produced without affecting N-glycans. Moreover, it was shown that changes in O-glycans directly influenced etanercept’s isoelectric point, TNF-α binding, and potency [75].

### 3.2. Enhancing Productivity and Cell Growth

Although enormous efforts have been put into cranking up yields of mAbs to more than 10 g/L, the demands of an increasing and highly competitive market still require production cell lines with even higher productivity [2]. On the other hand, the expression of novel therapeutic proteins with complex structures and achieving high-yield production of so-called difficult-to-express (DTE) proteins remains a challenge in CHO cells [24]. One of the possible targets of which modulation can lead to improved protein production is microRNAs (miRNAs) [78]. Several studies have shown that overexpression or knockdown of miRNA in CHO cells impacts cell performance [79,80,81,82]. The first such report about genomic KO of miRNA was published by Raab et al. [83]. Using CRISPR-Cas9-mediated KO of cgr-miR-744, which was previously identified as miRNA associated with poor productivity [80], led to increased antibody production in the batch process for approximately two-fold in cgr-miR-744 KO cell line compared to the control [83].

Apoptosis is a popular target to improve culture longevity and productivity as it is the major cause of death of CHO production lines [84]. Caspases are well-known executioners of apoptosis [85] and therefore an obvious target of choice for KO. Knowledge generated in other mammalian cells together with the report about siRNA-mediated co-downregulation of caspase-3 and caspase-7 in CHO cells, which led to increased cell viability and extension of culture longevity [86], motivated researchers to study the impact of caspase KO on CHO cell line performance. Disruption of the caspase-7 gene using a multiplex CRISPR system together with homology-independent targeted integration of a reporter gene was reported [87]. Using two sgRNAs targeting exons flanking the active site of the protein, the targeted genomic fragment was deleted and simultaneously the gene encoding the enhanced green fluorescent protein (EGFP) reporter was inserted. Interestingly, the authors reported that caspase-7 KO reduced cell proliferation, and contrary to the expectations, the caspase-7 KO cells were less resistant to apoptosis induced by sodium butyrate. It was concluded that caspase-7 may have a role in the cell cycle progression in CHO cells, making it a not ideal target for the prevention of apoptosis [87]. Another popular target for preventing apoptosis is the BCL-2 protein family and their CRISPR-Cas9 mediated KOs had a positive impact on CHO cell productivity and viability [88,89,90].

A further aspect to consider when trying to improve a production process is the secretory capacity of the producer cell line. In high-titer recombinant DTE protein production, the transcriptional supply can overwhelm downstream events such as protein folding, subunit assembly, post-translational modifications, and vesicular transport, thus in fact limiting production yield. Plasma cells, effector B lymphocytes specialized in antibody production, have an excellent secretory capacity and can modulate unfolded protein response (UPR), autophagy, and mTOR signaling pathways. Kim et al. [24] have expressed a master regulator responsible for the differentiation of B cells, B-lymphocyte-induced maturation protein-1 (Blimp1), in CHO cells in an attempt to induce cellular reprogramming and mitigate the limited secretion capacity. First, a recombination site was introduced in a predefined transcriptionally active locus using CRISPR-Cas9-mediated knock-in. Next, targeted integration of a single copy of the transcription factor Blimp1β-encoding gene was achieved via recombinase-catalyzed cassette exchange. Although Blimp1β somewhat suppressed cell growth, it enhanced the expression of a DTE protein, recombinant human bone morphogenetic protein-4, more than four-fold. Ectopic Blimp1β expression in CHO cells was found to elicit distinct gene expression patterns that promoted protein processing in secretory organelles.

To achieve high expression of recombinant proteins on an industrial scale, strong viral-derived promoters, e.g., CMV, are commonly utilized in cell line development [91]. However, several downsides are associated with exogenous promoters. Constitutive overexpression can lead to the activation of various regulatory mechanisms, such as unfolded proteins response (UPR) and endoplasmatic reticulum response, which can affect the correct processing of the recombinant protein or can lead to unstable production [91]. These problems can be overcome by using endogenous promoters. An interesting study reported the expression of a transgene in CHO cells via endogenous gene tagging [25]. The authors exploited the promoter of endogenous p21 to drive culture-dependent expression of the transgene encoding for the antiapoptosis effector protein human BCL-2. They integrated the *BCL-2* gene into the p21 locus using CRISPR-Cas9 and homology-directed repair and utilized its transcription regulation [25]. They synced BCL-2 with p21 expression during batch cultures, and induced p21 expression with a chemical inducer which led to a concomitant increase in BCL-2 expression, a drop in apoptotic activity, and extended culture longevity.

CHO genome plasticity enables valuable adaptability of these production cell lines to various genetic manipulation and changing process conditions. However, genome plasticity can also contribute to cell line instability and loss of productivity which is commonly observed in CHO production cell lines [92]. The causes for instability can either be due to the loss of transgene copies or transcription silencing [93]. For the latter, hypermethylation was detected in the CMV promoter region in low-producing CHO cells [94]. Therefore, regulating DNA methylation would be beneficial for long-term stable protein production. Jia et al. [18] tackled this by knocking out the *Dnmt3a* gene which encodes the protein involved in DNA methylation. Utilizing the CRISPR-Cas9 system, they disrupted the targeted gene sequence and consequently reduced its expression level in CHO cells leading to stable production that was maintained during long-term cultivation. Deducing that compromised DSB repair capacity is most likely the main reason for CHO genome instability has driven research published by Spahn et al. [95]. After performing a whole-genome sequencing of 11 different CHO cell lines and the native Chinese hamster genome, they found that CHO cell lines possess several mutations in DSB repair genes leading to DSB repair deficiency. They demonstrated that restoring the key DSB repair genes improves the repair capacity and genome instability of the CHO cell line. Moreover, overexpression of intact DNA repair genes *Xrcc6* and *Lig4* led to the improved stability of the transgene copy number and titer [95].

Although being widely adopted as an expression system, the CHO cell metabolism is far from optimal. It is characterized by high uptake rates of substrates, such as glucose and amino acids, which are diverted toward metabolic byproducts. Some of these byproducts can be cytotoxic, among which the most known are lactate and ammonium, and their accumulation negatively impacts cell growth, productivity, and product quality [96,97]. Typical targets for addressing this problem are glucose and glutamine metabolism [98]. Amino acid (AA) catabolism, which is directly linked to ammonium production and indirectly to lactate production is another cellular pathway that was reprogrammed in CHO cells [19]. It was previously shown that AA catabolism leads to the accumulation of not only lactate and ammonium, but also other growth-inhibiting compounds [97]. Ley et al. [19] used CRISPR-Cas9 to perform targeted disruption of various pathways involved in AA metabolism to ideally increase the availability of AA for proteogenesis and simultaneously reduce the synthesis of toxic byproducts. Disrupting two out of nine targeted genes, *Gapd2* and *Hpd*, yielded desired phenotypes, such as an increased growth rate, viable cell densities, and decreased specific lactate and ammonium secretion. Importantly, their data highlights the usefulness of cell line engineering strategies for improved CHO bioprocessing.

The UPR pathway is activated upon increased accumulation of unfolded or misfolded protein in the ER lumen, also known as ER stress. In the case of chronic ER stress, UPR induces apoptosis [99]. The increased production of recombinant proteins, especially complex DTE proteins in production cell lines, can trigger ER stress; therefore, real-time monitoring of the degree of UPR could be beneficial for achieving high productivity. The ER stress monitoring system created by using a CRISPR-Cas9-mediated targeted approach was developed in CHO cells. The authors showed that this could be achieved by monitoring the expression of the major ER chaperone BiP. Moreover, they showed that their monitoring system could be used as a screening strategy for the selection of high-producing CHO clones [100].

CRISPR-Cas9 is a versatile tool that can also be used as a validation technique for large-scale functional screens. Lin et al. [101] performed a large-scale siRNA screen to identify genes related to the productivity of CHO cells. This work distinguishes itself from previous siRNA-based screens in CHO cells [102] by using a CHO-specific siRNA library created by the authors. CRISPR-Cas9-mediated KO of four siRNA-identified genes was used to validate their role in productivity. A single knockout of three genes *Cyp1a2*, *Atp5s*, and *Dgki* resulted in a more than 90% increase in antibody productivity of the CHO cell line. Among them, the *Cyp1a2* gene showed the most promising results in productivity enhancement and its KO did not impair cell growth [101].

### 3.3. Tackling Adventitious Agents by CRISPR-Cas9

As pointed out in the introduction, one of the reasons for the extensive adoption of CHO cells in the biopharmaceutical industry is their reduced susceptibility to human viruses [103]. Nonetheless, there have been reported instances of viral contamination detected in manufacturing processes [104,105,106], and in all cases, the raw materials were suspected to be the source of viral contamination. A demonstration of freedom from adventitious viral agents is therefore one of the main regulatory requirements for biopharmaceutical approval [103]. Genome engineering tools can be utilized to enhance the safety of production cell lines, and in the following section we discuss different engineering approaches for viral resistance enhancement in CHO cells.

One of the strategies is targeting the sialylation pathway reported by Mascarenhas et al. [76]. To generate a host cell line resistant to mouse minute virus (MMV) infection, the authors used both ZFN and CRISPR-Cas9 technology to systemically KO genes involved in sialylation (*Slc35c1)* and two genes (*Mgat1* and *Cosmc)* impacting the major glycosylation types. It has been previously shown that MMV enters host cells through their surface sialoglycoprotein receptors [107]. Maccarenhas et al. [76] showed that KO of the *Slc35c1* gene leads to the complete absence of sialic acid on the cell line and consequently complete resistance to MVM infection, while KOs of *Cosmc* and *Mgat1* resulted in a significant decrease of infection.

Another possible strategy for preventing viral infection is by modulating innate immunity in CHO cells. For example, when the cells were treated with the immunomodulator polyinosinic:polycytidylic acid (poly I:C), a double-stranded RNA ligand of TLR3 receptor, STAT1-dependent regulatory network was induced, leading to a type I interferon response that protected cells from viral infection [20]. Using RNA-seq differential expression data, the authors then analyzed cellular responses after RNA virus infection vs. non-infected controls (with or without poly I:C treatment) to search for upstream STAT1 regulators that could be modulated to enhance viral resistance. Two negative regulators of STAT1, *Gfi1*, and *Trim24*, were identified in CHO cells. Knocking out the two genes with CRISPR-Cas9 confirmed their role in the suppression of a number of genes involved in innate immunity pathways, indicating that KO cells might possess enhanced immune functions. Indeed, in a proof-of-concept experiment in which the KO cells were challenged with RNA viruses, a notable increase in antiviral resistance was detected [20].

The third study addressing adventitious agents was published in 2020 by Duroy et al., in which type-C endogenous retrovirus (ERV) sequences in the CHO genome were investigated [21]. The presence of these sequences triggered safety concerns, although there is no direct evidence of their infectivity [21,108]. In the study, the authors discovered full-length transcripts with intact open reading frames from one C-type ERV group, indicating this particular group could produce functional viruses. Using CRISPR-Cas9 genome editing, they generated a number of mutations in the *gag* gene of the expressed ERV group, which lead to the identification of the ERV responsible for the release of RNA-loaded viral particles [21]. All three studies demonstrated that genome editing tools can be employed to minimize potential viral contamination in the production of biopharmaceuticals and consequently contribute to the safety of the final drug product.

### 3.4. CRISPR-Cas9-Mediated Elimination of Problematic Host Cell Proteins

Besides N-glycosylation, impurities represent an important class of drug substance CQAs, due to their potential impact on drug product safety [109]. Host cell proteins (HCPs) are process-related impurities that must be eliminated from the final drug product as they represent a potential risk for adverse immunological response in humans [110] or their impact the final drug product quality [111,112]. The majority of HCPs are usually removed during downstream processing, and only some of them are known as difficult to remove [113].

Lipoprotein lipase (LPL) is one of the problematic HCPs [22,114,115]. It has been hypothesized that LPL may degrade polysorbates which are typically used as excipients in the final drug formulation, negatively impacting drug product stability [22]. Therefore, LPL represents an ideal candidate for gene disruption or KO. CRISPR-Cas9-mediated KO of the LPL gene in CHO cells resulted in a more than 95% reduction of LPL expression and a reduction of polysorbate degradation by approximately 50% without affecting cell growth [22]. Fukuda et al. [116] investigated whether removing other potentially problematic HCPs impairs CHO cell growth. The authors chose three HCPs commonly detected in eluates after Protein A-affinity chromatography for KO; cathepsin D (*Ctsd*) as lysosomal protease impacting the structural integrity of mAbs, annexin A2 (*Anxa2*) as potentially immunogenic HCP [117], and calreticulin (*Calr*) as HCP currently not well understood [116]. CRISPR-mediated KOs of *Ctsd* and *Anxa2* genes were successfully generated, while on the other hand, the authors were not able to create KO of the *Calr* gene, despite several attempts. How these KOs impact the productivity of cognate cell lines has yet to be assessed, but so far it was observed that cell growth was not impaired and neither catepsin D nor annexin A2 were detected in the supernatants or lysates. Altogether, the results indicate such cell lines could be useful for manufacturing therapeutics with improved quality and safety profile. Other authors downregulated cathepsin D with shRNA technology and CRISPR-mediated KO in mAb-producing CHO cell lines [118]. Similar to the previous report [116], no impact on cell growth was observed during standard passaging in KO cell lines, however a decline in viability, viable cell density (VCD), and titer was observed in the fed-batch process. On the other hand, no negative impact on cell growth or productivity was seen in shRNA-treated clones, in which knockdown was efficient in minimizing HCP-related fragmentation to non-detectable levels [118].

Another study looked at proteolysis caused by HCP from the manufacturing of the HIV vaccine [119]. Specifically, the recombinant HIV envelope protein glycoprotein 120 (gp120), frequently used in HIV vaccines tested to date [120], is susceptible to proteolytic clipping by a serine protease. Due to the lack of CHO genome annotation, the identity of the responsible protease had been unknown for a long time [119,121]. Li et al. [119] identified the complement C1s subcomponent (C1s) serine protease, responsible for the clipping of recombinant gp120 expressed in CHO cells and showed that *C1s* KO prevents proteolysis. The beneficial impact of disrupting the N-glycan maturation in cell lines used for the production of HIV vaccines was reported previously [122], so Li et al. [119] generated CRISPR-Cas9-mediated KOs of *C1s* and alpha-1,3-mannosyl-glycoprotein 2-beta-*N*-acetylglucosaminyltransferase (*Mgat1*) in a stable gp120 expressing CHO cell line. This resulted in the production of unclipped gp120 with desired high mannose structures required for the binding of neutralizing antibodies. While this cell line was exclusively developed for expressing gp120, the same group later created two novel CHO cell lines suitable for expressing any type of therapeutic protein [120]; a *C1s*^−/−^ CHO cell line in which only the *C1s* gene is inactivated and a *C1s*^−/−^ *Mgat1*^−^ CHO cell line, where both *C1s* and *Mgat1* genes are inactivated. This work demonstrates how gene editing can overcome issues of protein clipping and glycosylation heterogeneity, and consequently accelerates HIV vaccine development.

The most comprehensive study to date on HCP removal in CHO cells reported the creation of multiplex CRISPR-Cas9-mediated KO of 6, 11, and 14 genes encoding for HCPs that were analyzed for total HCP content, cell productivity, and growth characteristics. Target genes selected for KO were either HCPs abundant in CHO supernatants, known to be difficult to remove with downstream processes or had an impact on product quality. A substantial reduction of total HCP level (40–70%) and increased productivity and cell growth were observed. This work demonstrates how to establish a «clean» CHO production cell line with superior performance by eliminating specific HCPs [123].

The heterogeneity of C-terminal lysine levels is commonly observed in monoclonal antibodies, and this reflects manufacturing and drug product consistency [124]. It was long speculated that one or more carboxypeptidases are involved in proteolysis, but it was unknown which one specifically is responsible for the removal of C-terminal lysine in CHO cells. Hu et al. [125] investigated the endogenous carboxypeptidases and their contribution to the variability of C-terminal lysine levels. The initial analysis of two distinct CHO host cell lines and two antibody-producing CHO cell lines revealed that among five different carboxypeptidases (CpD, CpM, CpN, CpB, and CpE), carboxypeptidase D (CpD) had the highest mRNA expression. The complete deletion of the CpD sequence in the IgG1 producing CHO cell line executed by CRISPR-Cas9 system using two sgRNAs targeting exon 1 and exon 21, respectively, abolished the antibody heavy chain C-terminal lysine removal. Mass spectrometry analysis showed up to 6% of C terminal lysine observed in wild-type clones, while 100% C-terminal lysine was detected in CpD KO clones. This clearly demonstrated that CpD is the only carboxypeptidase responsible for C-terminal lysine clipping in CHO cells [125].

**Table 1:** The summary of selected CRISPR KO studies in CHO cell line engineering.

Application	Target Gene	Gene Editing Method	Outcome	Reference
Fucosylation and formation of elongated glycans in O-glycosylation	*Fut8*, *Cosmc*	CRISPR-Cas9-mediated KO	Indel frequency of up to 47.3% in *Cosmc* and 42.5% in *Fut8.* Applying lectin selection, the frequency was improved by up to 99.7% in *Fut8*.	[57]
Fucosylation	*Fut8*	CRISPR-Cas9-mediated KO	Indel frequencies up to 25%, improved up to 52% with lectin selection.	[17]
Fucosylation	*Slc35c1*	ZFNs, TALENs, and CRISPR-Cas9-mediated KO	Cas9-mediated indel frequency up 18.4%; production of EPO-Fc fusion protein and anti-Her2 antibody without core fucose.	[58]
α-2,6-sialylation	*St3gal4*, st3gal6, *St6gal1*	CRISPR-Cas9-mediated KO and *St6gal1* overexpression	Recombinant IgG with predominantly α-2,6 sialylation.	[64]
NGNA sialylation	*Cmah*	CRISPR-Cas9-mediated KO	Complete loss of the NGNA sialylation on the IgG4 antibody.	[65]
Glycoengineering of alpha-1-antitrypsin and plasma protease C1 inhibitor	*KO: Mgat4A*, *Mgat4B*, *Mgat5*, *St3gal3*, *St3gal4*, *St3gal6*, *B3gnt2*, *Fut8*, *Sppl3*, and *Glul*; *OE*: ST6GAL1	CRISPR-Cas9-mediated KO and overexpression	Achieving glycosylation profile of recombinant proteins similar to the plasma-derived A1AT and C1INH.	[68]
Galactosylation	*B4galt1*, *B4galt2*, *B4galt3*, and *B4galt4*	Combinatorial CRISPR-Cas9-mediated KO	Reducing the levels of galactosylated N-glycans to ~6% and ~3% on transiently expressed erythropoietin (EPO) and rituximab from triple KO clone.	[71]
Glycoengineering options for lysosomal replacement enzymes	43 genes involved in N-glycosylation and mannose 6-phosphate processing	Individual and multiple CRISPR-Cas9-mediated KO	Improved circulation time and delivery to the heart of glycoengineered alpha-galactosidase in a Fabry disease mouse model.	[72]
Glycoengineering of therapeutic protein	19 glycosyltransferase genes controlling N-glycosylation	ZFNs, TALENs, and CRISPR-Cas9-mediated KO	Target changes in the glycosylation profile of EPO. Identified key glycogenes controlling steps in N-glycosylation of proteins in CHO cells.	[49]
O-glycosylation	A number of genes involved in O-glycosylation	CRISPR-Cas9-mediated KO	O-glycoengineered CHO cell line platform for the production of engineered proteins with desired O-glycans.	[75]
Improving productivity	cgr-miR-744	CRISPR-Cas9-mediated KO	Up to a 2-fold increase in antibody production.	[83]
Productivity	*Casp*-*7*	Multiplex CRISPR homology-independent target integration (HITI) with KO	KO of *Casp*-*7* lowered proliferation by up to 30% and reduced apoptosis resistance in KO clones.	[87]
Improving productivity and viability	*Bax*, *Bak*	CRISPR-Cas9-mediated double KO	Double KO clones with improved viability and up to 80% increase of productivity in intensified fed-batch.	[88]
Reducing apoptosis	*Bak1*, *Bax*, and *Bok*	Combinatorial CRISPR-Cas9-mediated KO	No detected impact on cell culture performance of *Bok* KO. Slower and delayed apoptosis in *Bak1* and *Bax* KOs.	[89]
Improving secretory capacity	*Blimp1*	CRISPR/Cas9-based recombinase-mediated KI	Up to 4-fold increased specific productivity of DTE recombinant human bone morphogenetic protein-4.	[24]
Reducing apoptosis	*BCL2*	CRISPR-Cas9-mediated knock-in	Integration of human *BCL2* into endogenous promoter locus reduced apoptosis.	[25]
Improving protein expression stability	*Dnmt3a*	CRISPR-Cas9-mediated KO	Enhanced long-term stability of transgene expression under CMV promoter.	[18]
Improving genome instability	CRISPR: *Atm*, *Prkdc*; OE: *Xrcc6* and *Lig4*	CRISPR-Cas9-mediated HDR-based gene correction and gene overexpression	DNA repair gene correction improved DNA repair and karyotypic instability. Overexpression of *Xrcc6* and *Lig4* led to improved stability of transgene copy number and productivity.	[95]
Reprogramming amino acid catabolism	*Aass*, *Afmid*, *Ddc*, *Gad1*, *Gad2*, *Prodh*, LOC100759874, *Gapd2* and *Hpd*	CRISPR-Cas9-mediated KO	KOs of *Gapd2 and Hpd* increased growth rates by up to 19%, VCDs up to 50%, and up to 26% and 22% decrease in specific ammonium and lactate production, respectively.	[19]
Monitoring ER stress	BiP	CRISPR-Cas9-mediated KI	Generation of monitoring system for UPR activation detection upon ER stress.	[100]
Improving productivity	*Cyp1a2*, *Atp5s*, and *Dgki*	CRISPR-Cas9-mediated KO	*Cyp1a2*, *Atp5s*, or *Dgki* KOs led to more than 90% increased specific antibody productivity.	[101]
Improving resistance to adventitious agents	*Slc35c1*, *Mgat1* and *Cosmc*	CRISPR-Cas9-mediated KO	*Slc35a1* KO led to complete resistance to MVM infection, while *Mgat1* and *Cosmc* KO led to significant inhibition of infection.	[76]
Improving resistance to adventitious agents	*Gfi1* and *Trim24*	CRISPR-Cas9-mediated KO	Increased antiviral resistance.	[20]
Eliminating viral particle contaminants	*Gag*	CRISPR-Cas9-mediated KO	Loss of function mutation in *Gag* gene led to reduced viral particle release.	[21]
Problematic HCP removal	*Lpl*	CRISPR-Cas9-mediated KO	Improved stability of PS20 (up to 57%) and PS80 (up to 47%) without significant impact on cell viability.	[22]
Problematic HCP removal	*Ctsd*, *Anxa2*, *Calr*	CRISPR-Cas9-mediated KO	*Ctsd* and *Anxa2* KOs led to complete elimination of corresponding HCPs in cell lysates without affecting cell growth and viability.	[116]
Problematic HCP removal	*Ctsd*	shRNA interference and CRISPR-mediated KO	*Ctsd* KO led to almost complete elimination of the associated proteolytic degradation in purified mAbs.	[118]
Problematic HCP removal and glycoengineering of cell lines for HIV vaccine production	*C1s*, *Mgat1*	CRISPR-Cas9-mediated KO	*C1s*/*Mgat1* KO led to production of unclipped gp120 protein with high mannose glycans.	[119,120]
HCP removal for protein production enhancement	*Timp1*, *Lgals3bp*, *Bgn*, *Nid1.1*, *Nid1.2*, *Ctsd*, *Tinagl1*, *Erp29*, *Aga*, *Lgmn*, *Gpr56*, *Yeats2*, *Sparc*, *Lpl*	Multiple CRISPR-Cas9-mediated KOs	6, 11 and 14 KOs led to 40–70% reduction of total HCP content and improved productivity and cell growth of selected clones.	[123]
Problematic HCP removal	*CpD*	CRISPR-Cas9-mediated KO	*CpD* KO led to complete abolishment of C-terminal lysine cleavage on IgG1.	[125]

KO (knockout); KI (knockin); OE (overexpression); VCD (viable cell density).

## 4. Toward Antibiotic-Free Recombinant Protein Production

While antibiotic-based selection is the most commonly used strategy to generate mammalian cells stably expressing a gene of interest (GOI) in laboratory settings [126], the biopharmaceutical industry typically utilizes auxotrophic selection systems based on either dihydrofolate reductase (DHFR) or glutamine synthetase (GS) with subsequent amplification with methotrexate (MTX) or methionine sulphoximine (MSX), respectively [127]. However, these approaches rely on the generation of KO cell lines deficient in essential enzymes. An alternative approach was reported by Teixeira et al. [126], who generated a selection system, named CelloSelect, in which cells growing in glucose-free media start metabolizing cellobiose as a primary source of energy. Here, the selection cassette contained genes encoding *Neurospora crassa* cellodextrin transporter CDT1 and beta-glucosidase GH1-1. This technology allows selection without generating KO cell lines and omits using toxic antibiotics or small molecules such as MTX or MSX. Another CHO production cell line allowing antibiotic-free selection was developed by using CRISPR-Cas9 mediated KO of 10 genes involved in the purine/pyrimidine biosynthetic pathway [128]. This multiauxotrophic cell line enables the selection of clones expressing up to eight different transgenes after a single transfection without any antibiotics or other selection markers.

## 5. From Random Integration toward CRISPR-Cas9-Mediated Site-Specific Knock-In

Typical CHO cell line development starts with the random integration of a recombinant protein-expressing plasmid into the host, subsequent selection of stable CHO cell pools, and lastly the generation of stable clones [129]. Random integration of such plasmids results in clones with variable integration sites and copy numbers, which consequently leads to clones with variable productivity. Usually, an extensive clone screening is necessary to identify clones with sufficiently high and stable productivity, and other quality characteristics [129]. Clones with high productivity are often susceptible to production instability resulting from epigenetic silencing or loss of transgene copy number due to chromosomal rearrangements and deletions [129,130]. The site-specific integration (SSI) approach offers a solution to avoid random integration into unstable genomic regions by inserting transgenes into predefined transcriptionally active and stable genomic loci, so-called «hot-spots» [129,130]. Several potential hot-spots were identified in CHO cells, however, only a few of them were validated in an industrial setup [130,131,132]. Large regulatory elements called super-enhancers are accessible genomic regions [133] favoring stable and highly active transcription, which could be ideal for the integration of genomic recombinase recognition sites, referred to as landing pads [26,129,134]. Several different approaches of SSI using site-specific recombinases, such as the Cre/loxP system, Flp/FRT system, phiC31/R4, and Bxb1 integrases, were tested in CHO cells [129]. However, the drawback of recombination-based systems is the required preestablishment of master cell lines with landing pads [26,129]. Available CHO genome sequence and the development of targetable nucleases enabled nuclease-mediated SSI or desired sequence changes [27,129]. Similar to gene KO, nuclease-mediated SSI utilizes DSBs. The following transgene integration could be achieved by different DNA repair mechanisms, among which the most commonly used is homology-directed repair [129]. The subsequent chapter includes a summary of different CRISPR-Cas9-mediated site-specific transgene integration approaches studied in CHO cells.

Numerous studies described combining recombinase- with nuclease-mediated site-specific integration. Inniss et al. [135] compared two different recombinase-mediated cassette exchange (RMCE) systems inserted to a specific locus in the CHO genome by CRISPR-Cas9 site-specific integration with a homology-directed repair. They showed that the BxB1 integrase system yielded higher fidelity RMCE events and represents a great alternative to the more established Flp/FRT system. Others reported on the development of a highly efficient system for site-specific integration based on the combination of CRISPR-Cas9-mediated SSI with bacteriophage PhiC31 integrase [136]. Another CRISPR-Cas9-based tool for targeted integration was developed by Pristovšek et al. [26], who created a modular toolbox for the construction of mammalian cell lines with targeted integration of a landing pad, possessing a recombinant gene under defined 5′ proximal regulatory elements. They set out to study different expression cassette designs in newly-discovered safe harbors in CHO cell lines and demonstrated that high and/or stable expression levels in defined chromosomal loci are restricted to a specific cassette design. Their toolbox was used by Sergeeva et al. [137], who modified it in a way that allowed for a multicopy target integration at a single site, and the simultaneous integration in two genomic sites. They showed that a transcriptional bottleneck can appear when the copy number is increased (>2 copies) at a single genomic site. Their dual-RMCE system sped up the generation of CHO production cell lines with high productivity and high titers, making it suitable for the needs in the industrial production of therapeutic proteins [137].

Other authors report using the CRISPR-Cas9 system and homology-directed repair for direct site-specific integration in CHO cells (i.e., without an additional recombinase-catalyzed step). The CRISPR-Cas9 editing system with donor plasmid possessing homology arms and a GOI was used to insert a large gene expression cassette at three loci in the CHO genome (*Cosmc*-, *Mgat1*-, and *LdhA*-encoding sites) with a targeting efficiency between 7.4–27.8%, depending on the target locus and CRISPR-Cas9 activity [27]. A follow-on paper reported on improved HDR-mediated integration with an antibiotic-free selection approach. Two ways to enhance HDR efficiency were tested, e.g., chemical treatment and FACS enrichment. While the chemical treatment with the DNA ligase IV inhibitor Scr7 or lithium chloride had no significant effect on efficiency, fluorescent enrichment resulted in a 3-fold increase in the number of cells with HDR-mediated genome editing [138].

To summarize, the results of all mentioned studies indicate that site-specific integration of transgene outperforms the conventional way (i.e., random integration) of gene integration in CHO cells. Moreover, it was shown that these approaches are successful in the targeted integration of large protein-encoding genes (including antibodies) and greatly accelerate generating new CHO production cell lines.

## 6. Applications of CRISPR-Mediated Gene Activation and Repression in CHO Cells

The initial demonstration of the simplicity and effectiveness of the CRISPR-Cas9-based genome editing was soon followed by the modification of the CRISPR platform for regulating transcription [41]. First studies using mutant Cas9, known as «dead» Cas9 (dCas9) [42,43], lacking endonuclease activity but retaining selective DNA binding ability, as specified by sgRNA, demonstrated that transcription of targeted genes can be modified without altering the targeted genomic sequence. A catalytically inactive version of Cas9 homing a target gene region can suppress its transcription by sterically obstructing RNA polymerase binding or elongation, leading to strong repression in bacteria, and modest repression in human cells [43]. Succeeding studies showed that dCas9 can be fused with different previously established transcription activators (e.g., VP64, VPR [139]) or repressors (e.g., KRAB [140]) enabling strong gene activation or repression, respectively, in human cells [140,141]. A detailed overview of different dCas9 variants and applications was comprehensively described in several review papers [41,142].

The first demonstration of CRISPR interference (CRISPRi) technology in CHO cells was reported by Shen et al. [31], who utilized it to enhance protein production. They used dCas9 fused to the KRAB transcription repression domain to suppress *Dhfr* transcription. *Dhfr* gene is commonly cotransfected with a GOI enabling typical DHFR/MTX selection for co-amplification of *Dhfr* and the GOI in CHO cells, leading to enhanced GOI expression levels. Their approach imposed additional selective pressure forcing cells to co-amplify more copies of *Dhfr* and the adjacent GOI. This resulted in a 3.8-fold increase in protein expression in the case of EGFP and a 2.8-fold increase in the case of a pharmaceutical protein granulocyte colony-stimulating factor. In short, Shen et al. [31] were the first to show CRISPRi technology can be used to enhance recombinant protein production in CHO cells. A year later, a study demonstrating endogenous gene repression using CRISPRi in CHO cells was published, where three genes involved in apoptosis, *Bak*, *Bax*, and *Casp3*, were targeted [32]. This led to reduced caspase activity and apoptosis, and improved mitochondrial integrity. An important finding of this study is that repression efficiencies of the CRISPRi system can be enhanced by different repressor fusion types, e.g., N-terminal or C-terminal. They observed better efficiency of C-terminal KRAB fusion, contrary to the reports from human cells [141]. Additionally, the impact of bipartite repressor type dCas9-KRAB-Mecp2, with previously reported superior repression efficiency in human cells [143], was evaluated and concluded that an additional repressor can lead to improved CRISPRi regulation efficiency in gene repression in CHO cells [32]. Both studies reporting CRISPRi in CHO cells used KRAB repressor fused to the Cas9 originating from *S. pyogenes.* In our latest study [33], we were the first to report exploring the CRISPRi system in which KRAB was fused to dCas9 originating from *Staphylococcus aureus* in CHO cells. *S. aureus* Cas9 recognizes a different PAM (5′-NNGRRT-3′ (where R represents A or G)) [144]. It showed comparable gene editing efficiency to SpCas9 in other mammalian cell lines, but its smaller size (1053 amino acids compared to 1368 amino acids of SpCas9) makes it applicable for size-restricted plasmid delivery [144,145]. We demonstrated that CRISPRi gene repression can be enhanced when coupled with lectin-based FACS enrichment of cells with low surface fucosylation. We believe such an approach can be very useful for developing biosimilars.

The discovery of type IV CRISPR-Cas systems based on Cas13 opened the possibility for CRISPR-based RNA targeting [146]. These systems utilize the protein effector Cas13 guided by a single RNA to target a specific RNA molecule [146]. Several different effectors were discovered in the bacterial genome (Cas13a, Cas13b, and Cas13d) and Cas13d was reported as the most effective in RNA knockdown [144,146,147]. The Cas13d variant is one of the smallest Cas proteins discovered so far (930 amino acids in length) [148], and, in contrast to Cas9 and Cas12a, targets RNA which makes it suitable for the modulation of gene expression without disrupting the genome and may serve as an alternative to dCas9 variants [149]. It was also shown that this system can efficiently knock down exogenous and endogenous genes involved in various cellular pathways, such as apoptosis, metabolism, gene amplification, and glycosylation in CHO cells [35,36].

The first application of targeted CRISPR-based activation of an endogenous gene in CHO cells was reported by Marx et al. [28], where they targeted the silenced *St6gal1* gene. The activation was achieved by fusing dCas9 to the catalytic domain (CD) of the ten-eleven translocation methylcytosine dioxygenase 1 (TET1), guided to the *St6gal1* promoter region, to target demethylation. Furthermore, they reversed this effect by targeting CD of DNA methyltransferase (DNMT) to re-methylate the promoter [28]. In a further study, the same epigenetic tool was applied to modulate the epigenetic status of the exogenous CMV promoter, on endogenous, natively silenced *St6gal1*, and the natively active *Fut8* promoter [29].

Beta-galactoside alpha-2,6-sialyltransferase 1 together with another silenced gene, *Mgat3*, was chosen for upregulation in a study published by Karottki et al. [30]. α2,6-linked sialic acid and bisecting GlcNAc motifs commonly decorate human glycoproteins but are absent from proteins expressed by CHO cells. Hence, upregulating these two genes in CHO cells could yield therapeutic proteins with a more human-like glycosylation pattern. The authors fused dCas9 with one of the most potent transcription activator domains VPR [150]. In contrast with TET fusion, which needs to be guided to methylated promoter regions, a tool known as CRISPR activation (CRISPRa) should in principle be guided to a region upstream of the transcription start site (TSS) and achieve upregulation of endogenous gene expression [141]. Since the TSSs of target genes had not yet been experimentally determined, Karottki et al. [30] relied on the CHO-K1 NCBI annotation database to design multiple sgRNAs and successfully induced upregulation at the mRNA level. Glycan analysis comparing transfected CHO cells to an untransfected control detected glycans with bisecting GlcNAc and α-2,6 sialic acid but the upregulation was modest. An important takeaway is that if CHO genes are computationally predicted and information about TSS is absent, multiple sgRNAs need to be designed and tested to find a functional one if any at all.

The issue of poor CHO genome annotation was tackled by the same researchers [151]. They applied multiple complementary RNA sequencing methods to analyze 10 different Chinese hamster tissues, bone marrow-derived macrophages, and the CHO K1 cell line to accurately determine TSSs. More than 70% of annotated CHO genes and non-coding RNAs, including many silenced genes, were mapped and realigned to existing RefSeq TSSs, which were found to be incorrect. Additionally, it was shown that revised annotations facilitate activation of a normally dormant gene *Mgat3* using CRISPRa, although the newly identified TSS of the *Mgat3* gene is >25 kb upstream of the RefSeq TSS targeted in their previous study [30]. The data obtained during this study provides essential information for CHO cell line engineering and a rich resource for future research and development of production cell lines.

## 7. CRISPR for Studying Gene Function in CHO Cells

The most widely used approach to study gene function is to repress or completely disrupt its expression. The discovery of RNA interference (RNAi) enabled silencing of specific genes and became the method of choice for deciphering gene function [12]. RNAi-based screening studies were performed in CHO cells to identify gene knockdowns, leading to desired phenotypic traits [101,102]. However, the inability to produce full knockouts and high off-target activity [152] hindered the improvement of RNAi technology. Traditional RNAi-based screens were gradually replaced by CRISPR-Cas9 technologies in large-scale screening studies [12]. In the past years, such screening studies were conducted on several mammalian cell lines [153] and recently also in CHO cells. Karottki et al. [152] performed a large-scale CHO-specific CRISPR-Cas9 KO screen targeting CHO cell metabolism. Their study gained new insight on genes involved in glutamine metabolism. It also revealed the gene *Abhd11* encoding for a lipase with no clear connection to glutamine action, which, when knocked-out, increased growth in glutamine-free media by altering the regulation of the tricarboxylic acid cycle. In the same year, two studies using CRISPR screening platforms were published, both reporting CRISPR screens without using lentiviral delivery set-ups [154,155]. This would be applicable for industrial set-ups, where such tools would allow searching for genes that enhance cellular features specific for biopharmaceutical needs.

The majority of the above studies utilized the approach of inducing single double-strand breaks, leading to frameshift mutations that result in gene disruption. However, indels do not always result in a frameshift, and frameshift mutations can still break away from nonsense-mediated decay. Moreover, there is a potential risk that altered transcripts could make up or change gene function. Genomic deletions created by using two sgRNAs to produce double DSBs (which leads to the loss of the intervening DNA) represent an alternative approach, especially useful in studies of protein function, where complete removal of proteins is necessary [156]. An example of such genomic deletion using the CRISPR system in CHO cells was reported in a study where gene deletion was coupled with a multiplexing approach to achieve the removal of FUT8 promoter and deletions in B4GALT1 isozyme genes [23]. This study is the first to report on Cas12a (previously termed Cpf1) activity in CHO cells. Like CRISPR-Cas9, Cas12a belongs to class 2 CRISPR-Cas systems and features a single-protein effector module [157,158]. However, Cas12a differs from Cas9 in several aspects. It is smaller compared to Cas9, it does not require tracrRNA for target cleavage, it cleaves DNA targets adjacent to T-rich PAM (while Cas9 recognizes G-rich PAM) and therefore expands the target sites, and lastly, it introduces a staggered double-stranded break with a 4- or 5-nt 5′ overhang [157,159]. The production of a staggered end may be an advantage for gene KI applications, where the orientation of the transgene is important [144]. As demonstrated by the authors, Cas9 and Cas12a can be efficiently used in parallel without crosstalk between distinct CRISPR systems in CHO cells [23]. Since then, several studies reported on CRISPR-Cas12a-mediated genome editing, confirming its applicability in CHO genome engineering [154,160,161].

## 8. Major Challenges of CRISPR-Cas in CHO Cell Line Engineering and Potential Future Directions

The majority of studies discussed in this review relied on naturally occurring CRISPR-Cas variants for genome editing. Although being efficient at knocking-out targeted genes, some studies reported certain non-desired effects, such as impaired cell growth characteristics [24,83,101,118]. One of the possible reasons could be the off-target effects of utilized Cas9 nuclease which is commonly observed in other mammalian cell lines [162]. Several different approaches can overcome this issue. The use of a preassembled Cas9 and sgRNA complex in CHO cells reported by Lee et al. [163] offers an alternative approach with natural Cas9 variants but with minimized risk of random integration into unwanted genome regions together with reduced off-target effects [164]. Recent developments of synthetic Cas variants, such as CRISPR base editors, provide CRISPR tools with enhanced precision in editing DNA. CRISPR base editors represent a combination of catalytically impaired Cas nuclease, gRNA, and a base modification enzyme operating on single-stranded DNA without generating DSBs [165]. Although not yet extensively used in CHO cell line engineering, the reported improved efficiency and lower cytotoxicity of Cas9 nickase and dCas9 PmCDA1-mediated base editing compared to Cas9 KO [34] may lead to the broader exploitation of this approach in CHO cell line engineering in the future. Moreover, the CRISPR toolbox in CHO cell line engineering has recently been expanded to include the engineered Cas variants Mad7, also known as ErCas12a, in CHO cells [166].

The increasing complexity of therapeutic proteins requires an enhanced performance of expression systems. Therefore, the development of systems allowing efficient modulation of multiple cellular pathways is foreseen in CHO cell line engineering. Simultaneous use of orthogonal Cas variants possessing different PAM sequence requirements, as shown by Schmieder et al. [23], will likely broaden the number of genes targeted within the same cell. Moreover, Cas12a is capable of processing its pre-crRNA, making it suitable for multiplexed genome editing and genome screening [154,167]. Similarly, RNA ribonuclease Cas13 can process crRNA array making it applicable for multiplex gene knockdown [36]. Approaches allowing simultaneous modulation of gene expression in different directions were not yet tested in CHO cells but have been proven as a promising tool for transcription engineering in other mammalian cell lines [168]. Lastly, the availability of more accurate CHO genome annotations [169,170] together with the more precise engineered Cas variants [171,172,173] could simplify the search for efficient sgRNAs with minimized off-target effects in the CHO cell line engineering.

## 9. Conclusions

Our review aims to enlighten the progress made in CHO cell line engineering since the first application of the CRISPR technology in this mammalian host accommodated by a multi-billion dollars industry. As seen in other mammalian cell lines, the CRISPR systems proved to be a versatile tool allowing for simple, efficient, and affordable manipulation of the CHO genome. Both academia and the biopharmaceutical industry are facing ever more challenging tasks that are coming together with increasing demands to develop complex therapeutic proteins requiring production cell lines with specific modifications. New insights in genomics in general, with annotations of the CHO genome, have helped researchers successfully overcome obstacles in CHO cell engineering [169,170], but there are still data gaps for efficient sgRNA design. Advances in engineering therapeutic proteins, and modulating and editing gene expression, will enable easier production of DTE proteins with specific glycopatterns [174,175]. An increasing number of approved biosimilars in the EU and US markets [4] is contributing to the pace of innovation and development of innovative biologics and biosimilars. The availability of easy-to-use and precise CRISPR tools could play a key role in addressing future challenges in the development of biopharmaceuticals.

## Data Availability

No new data were created or analyzed in this study. Data sharing is not applicable to this article.

## Abbreviations

A1AT (α1-antitrypsin); AA (amino acid); AAL (*Aleuria aurantia* lectin); AATD (α1-antitrypsin deficiency); ADCC (antibody-dependent cellular cytotoxicity); *Anxa2* (annexin A2); *B3gnt2* (UDP-GlcNAc: betaGal beta-1,3-*N*-acetylglucosaminyltransferase 2); *B4galt* (beta-1,4-galactosyltransferase); *Bak1* (Bcl-2 homologous antagonist/killer); *Bax* (Bcl-2 associated X); C1INH (C1 esterase inhibitor); BLC2 (B-cell lymphoma 2); Blimp1 (B-lymphocyte-induced maturation protein-1); *Calr* (calreticulin); Cas (CRISPR-associated); *Casp3* (caspase 3); CD (catalytic domain); CHO (Chinese hamster ovary); *Cmah* (cytidine monophosphate (CMP)-*N*-acetylneuraminic acid hydroxylase); CMV (cytomegalovirus); *Cosmc* (C1GALT1-specific chaperone 1); Cp (carboxypeptidase); CQA (critical quality attribute); CRISPR (clustered regularly interspaced short palindromic repeat); CRISPRa (CRISPR activation); CRISPRi (CRISPR interference); crRNA (CRISPR RNA); *Ctsd* (cathepsin D); dCas13 (dead Cas13); dCas9 (dead Cas9); *Ddit3* (DNA damage inducible transcript 3); *Dhfr* (dihydrofolate reductase); *Dnmt3a* (DNA (cytosine-5)-methyltransferase 3A); DSB (double strand break); DTE (difficult-to-express); EGFP (enhanced green fluorescent protein); ERV (endogenous retrovirus); Fab (fragment antigen-binding); FACS (fluorescence-activated cell sorting); Fc (fragment crystallizable); *Fut8* (alpha-(1,6)-fucosyltransferase); *Gad2* (glutamate decarboxylase 2); *Gapd2* (glyceraldehyde-3-phosphate dehydrogenase 2); *Gfi1* (growth factor independent 1 transcriptional repressor); *Gft* (GDP-fucose transporter); GlcNAc (*N*-acetylglucosamine); *Glul* (glutamine synthetase); GOI (gene of interest); gp120 (glycoprotein 120); HCP (host cell protein); HDR (homology-directed repair); *Hpd* (4-hydroxyphenylpyruvate dioxygenase); *Hprt* (hypoxanthine phosphoribosyltransferase 1); KO (knock-out); KRAB (Krüppel associated box); LCA (*Lens culinaris* agglutinin); *Ldha* (lactate dehydrogenase-A); *Lig4* (DNA ligase 4); *Lpl* (lipoprotein lipase); LSD (lysosomal storage disease); mAb (monoclonal antibody); *Mgat1* (alpha-1,3-mannosyl-glycoprotein 2-beta-*N*-acetylglucosaminyltransferase); *Mgat3* (beta-1,4-mannosyl-glycoprotein 4-beta-*N*-acetylglucosaminyltransferase); *Mgat4a* (alpha-1,3-mannosyl-glycoprotein 4-beta-*N*-acetylglucosaminyltransferase A); *Mgat4b* (alpha-1,3-mannosyl-glycoprotein 4-beta-*N*-acetylglucosaminyltransferase B); *Mgat5* (alpha-1,6-mannosyl-glycoprotein 6-beta-*N*-acetylglucosaminyltransferase A); miRNA (micro RNA); MMV (mouse minute virus); MSX (methionine sulphoximine); MTX (methotrexate); NANA (*N*-acetylneuraminic acid); NGNA (*N*-glycolylneuraminic acid); PAM (protospacer adjacent motif); PmCDA1 (*Petromyzon marinus* cytidine deaminase 1); RGENs (RNA-guided engineered nucleases); RMCE (recombinase-mediated cassette exchange); sgRNA (single guide RNA); *Slc35c1* (GDP-fucose transporter); *Sppl3* (signal peptide peptidase-like 3); SSI (site-specific integration); *St3gal3* (CMP-*N*-acetylneuraminate-beta-1,4-galactoside alpha-2,3-sialyltransferase); *St3gal4* (CMP-*N*-acetylneuraminate-beta-galactosamide-alpha-2,3-sialyltransferase 4); *St3gal6* (type 2 lactosamine alpha-2,3-sialyltransferase); *St6gal1* (beta-galactoside alpha-2,6-sialyltransferase 1); TALENs (transcription activator-like effector nucleases); *Tet1* (methylcytosine dioxygenase ten-eleven translocation 1); tracRNA (transactivating RNA); *Trim24* (tripartite motif containing 24); TSS (transcription start site); UPR (unfolded protein response); *Xrcc6* (X-ray repair cross complementing 6); ZFNs (zinc-finger nucleases).

## References

[1] Fischer S., Handrick R., Otte K. (2015). The art of cho cell engineering: A comprehensive retrospect and future perspectives. Biotechnol. Adv..

[2] Kim J.Y., Kim Y.G., Lee G.M. (2012). CHO cells in biotechnology for production of recombinant proteins: Current state and further potential. Appl. Microbiol. Biotechnol..

[3] Jayapal K.P., Wlaschin K.F., Hu W.S., Yap M.G.S. (2007). Recombinant protein therapeutics from CHO cells—20 years and counting. Chem. Eng. Prog..

[4] Walsh G. (2018). Biopharmaceutical benchmarks 2018. Nat. Biotechnol..

[5] Datta P., Linhardt R.J., Sharfstein S.T. (2013). An’omics approach towards CHO cell engineering. Biotechnol. Bioeng..

[6] Walsh G., Walsh E. (2022). Biopharmaceutical benchmarks 2022. Nat. Biotechnol..

[7] Kwang Hong J., Lakshmanan M., Goudar C., Lee D.Y., Betenbaugh M., Titchener-Hooker N. (2018). Towards next generation CHO cell line development and engineering by systems approaches. Curr. Opin. Chem. Eng..

[8] Xu X., Nagarajan H., Lewis N.E., Pan S., Cai Z., Liu X., Chen W., Xie M., Wang W., Hammond S. (2011). The genomic sequence of the Chinese hamster ovary (CHO)-K1 cell line. Nat. Biotechnol..

[9] Lonowski L.A., Narimatsu Y., Riaz A., Delay C.E., Yang Z., Niola F., Duda K., Ober E.A., Clausen H., Wandall H.H. (2017). Genome editing using FACS enrichment of nuclease-expressing cells and indel detection by amplicon analysis. Nat. Protoc..

[10] Carroll D. (2014). Genome engineering with targetable nucleases. Annu. Rev. Biochem..

[11] Kim H., Kim J.S. (2014). A guide to genome engineering with programmable nucleases. Nat. Rev. Genet..

[12] Boettcher M., McManus M.T. (2015). Choosing the right tool for the job: RNAi, TALEN, or CRISPR. Mol. Cell..

[13] Sander J.D., Joung J.K. (2014). CRISPR-Cas systems for editing, regulating and targeting genomes. Nat. Biotechnol..

[14] Sanjana N.E., Cong L., Zhou Y., Cunniff M.M., Feng G., Zhang F. (2012). A transcription activator-like effector toolbox for genome engineering. Nat. Protoc..

[15] Urnov F.D., Rebar E.J., Holmes M.C., Zhang H.S., Gregory P.D. (2010). Genome editing with engineered zinc finger nucleases. Nat. Rev. Genet..

[16] Ferreira P., Choupina A.B. (2022). CRISPR/Cas9 a simple, inexpensive and effective technique for gene editing. Mol. Biol. Rep..

[17] Sun T., Li C., Han L., Jiang H., Xie Y., Zhang B., Qian X., Lu H., Zhu J. (2015). Functional knockout of FUT8 in Chinese hamster ovary cells using CRISPR/Cas9 to produce a defucosylated antibody. Eng. Life Sci..

[18] Jia Y., Guo X., Lu J., Wang X., Qiu L., Wang T. (2018). CRISPR/Cas9-mediated gene knockout for DNA methyltransferase Dnmt3a in CHO cells displays enhanced transgenic expression and long-term stability. J. Cell. Mol. Med..

[19] Ley D., Pereira S., Pedersen L.E., Arnsdorf J., Hefzi H., Davy A.M., Ha T.K., Wulff T., Kildegaard H.F., Andersen M.R. (2019). Reprogramming AA catabolism in CHO cells with CRISPR/Cas9 genome editing improves cell growth and reduces byproduct secretion. Metab. Eng..

[20] Chiang A.W.T., Li S., Kellman B.P., Chattopadhyay G., Zhang Y., Kuo C.C., Gutierrez J.M., Ghazi F., Schmeisser H., Ménard P. (2019). Combating viral contaminants in CHO cells by engineering innate immunity. Sci. Rep..

[21] Duroy P., Bosshard S., Schmid-Siegert E., Neuenschwander S., Arib G., Lemercier P., Masterneak J., Roesch L., Buron F., Girod P. (2020). Characterization and mutagenesis of Chinese hamster ovary cells endogenous retroviruses to inactivate viral particle release. Biotechnol. Bioeng..

[22] Chiu J., Valente K.N., Levy N.E., Min L., Lenhoff A.M., Lee K.H. (2017). Knockout of a difficult-to-remove CHO host cell protein, lipoprotein lipase, for improved polysorbate stability in monoclonal antibody formulations. Biotechnol. Bioeng..

[23] Schmieder V., Bydlinski N., Strasser R., Baumann M., Kildegaard H.F., Jadhav V., Borth N. (2018). Enhanced genome editing tools for multi-gene deletion knock-out approaches using paired CRISPR sgRNAs in CHO cells. Biotechnol. J..

[24] Kim S.H., Baek M., Park S., Shin S., Lee J.S., Lee G.M. (2022). Improving the secretory capacity of CHO producer cells: The effect of controlled blimp1 expression, a master transcription factor for plasma cells. Metab. Eng..

[25] Lee Y., Kwak J.M., Lee J.S. (2020). Endogenous p21-dependent transgene control for CHO cell engineering. ACS Synth. Biol..

[26] Pristovšek N., Nallapareddy S., Grav L.M., Hefzi H., Lewis N.E., Rugbjerg P., Hansen H.G., Lee G.M., Andersen M.R., Faustrup Kildegaard H. (2019). Systematic evaluation of site-specific recombinant gene expression for programmable mammalian cell engineering. ACS Synth. Biol..

[27] Lee J.S., Kallehauge T.B., Pedersen L.E., Kildegaard H.F. (2015). Site-specific integration in CHO cells mediated by CRISPR/Cas9 and homology-directed DNA repair pathway. Sci. Rep..

[28] Marx N., Grünwald-Gruber C., Bydlinski N., Dhiman H., Ngoc Nguyen L., Klanert G., Borth N. (2018). CRISPR-based targeted epigenetic editing enables gene expression modulation of the silenced beta-galactoside alpha-2,6-sialyltransferase 1 in CHO cells. Biotechnol. J..

[29] Marx N., Dhiman H., Schmieder V., Freire C.M., Nguyen L.N., Klanert G., Borth N. (2021). Enhanced targeted DNA methylation of the CMV and endogenous promoters with dCas9-DNMT3A3L entails distinct subsequent histone modification changes in CHO cells. Metab. Eng..

[30] Karottki K.J.L.C., Hefzi H., Xiong K., Shamie I., Hansen A.H., Li S., Pedersen L.E., Li S., Lee J.S., Lee G.M. (2020). Awakening dormant glycosyltransferases in CHO cells with CRISPRa. Biotechnol. Bioeng..

[31] Shen C.C., Sung L.Y., Lin S.Y., Lin M.W., Hu Y.C. (2017). Enhancing protein production yield from Chinese hamster ovary cells by CRISPR interference. Cells.

[32] Xiong K., Marquart K.F., la Cour Karottki K.J., Li S., Shamie I., Lee J.S., Gerling S., Yeo N.C., Chavez A., Lee G.M. (2019). Reduced apoptosis in Chinese hamster ovary cells via optimized CRISPR interference. Biotechnol. Bioeng..

[33] Glinšek K., Kramer L., Krajnc A., Kranjc E., Pirher N., Marušič J., Hellmann L., Podobnik B., Štrukelj B., Ausländer D. (2022). Coupling CRISPR interference with FACS enrichment: New approach in glycoengineering of CHO cell lines for therapeutic glycoprotein production. Biotechnol. J..

[34] Nishida K., Arazoe T., Yachie N., Banno S., Kakimoto M., Tabata M., Mochizuki M., Miyabe A., Araki M., Hara K.Y. (2016). Targeted nucleotide editing using hybrid prokaryotic and vertebrate adaptive immune systems. Science.

[35] Shen C.C., Lin M.W., Nguyen B.K.T., Chang C.W., Shih J.R., Nguyen M.T.T., Chang Y.H., Hu Y.C. (2020). CRISPR-Cas13d for gene knockdown and engineering of CHO cells. ACS Synth. Biol..

[36] Lin M.W., Shen C.C., Lin Y.J., Chou M.Y., Pham N.N., Chang Y.H., Chang C.W., Hwu J.R., Nguyen M.T.T., Hu Y.C. (2021). Enhancing the yield and activity of defucosylated antibody produced by CHO-K1 cells using Cas13d-mediated multiplex gene targeting. J. Taiwan Inst. Chem. Eng..

[37] Jinek M., East A., Cheng A., Lin S., Ma E., Doudna J. (2013). RNA-programmed genome editing in human cells. eLife.

[38] Cong L., Ran F.A., Cox D., Lin S., Barretto R., Habib N., Hsu P.D., Wu X., Jiang W., Marraffini L.A. (2013). Multiplex genome engineering using CRISPR/Cas systems. Science.

[39] Mali P., Yang L., Esvelt K.M., Aach J., Guell M., DiCarlo J.E., Norville J.E., Church G.M. (2013). RNA-guided human genome engineering via Cas9. Science.

[40] Doudna J.A., Charpentier E. (2014). The new frontier of genome engineering with CRISPR-Cas9. Science.

[41] Vora S., Tuttle M., Cheng J., Church G. (2016). Next stop for the CRISPR revolution: RNA-guided epigenetic regulators. FEBS J..

[42] Mali P., Aach J., Stranges P.B., Esvelt K.M., Moosburner M., Kosuri S., Yang L., Church G.M. (2013). CAS9 transcriptional activators for target specificity screening and paired nickases for cooperative genome engineering. Nat. Biotechnol..

[43] Qi L.S., Larson M.H., Gilbert L.A., Doudna J.A., Weissman J.S., Arkin A.P., Lim W.A. (2013). Repurposing CRISPR as an RNA-guided platform for sequence-specific control of gene expression. Cell.

[44] Walsh G. (2010). Post-translational modifications of protein biopharmaceuticals. Drug Discov. Today.

[45] Walsh G., Jefferis R. (2006). Post-translational modifications in the context of therapeutic proteins. Nat. Biotechnol..

[46] Delobel A. (2021). Glycosylation of therapeutic proteins: A critical quality attribute. Methods Mol. Biol..

[47] Tejwani V., Andersen M.R., Nam J.H., Sharfstein S.T. (2018). Glycoengineering in CHO cells: Advances in systems biology. Biotechnol. J..

[48] Schulz M.A., Tian W., Mao Y., Van Coillie J., Sun L., Larsen J.S., Andersen M.R., Chin P.T.K., Andersen M.R., Kildegaard H.F. (2018). Glycoengineering design options for IgG1 in CHO cells using precise gene editing. Glycobiology.

[49] Yang Z., Wang S., Halim A., Schulz M.A., Frodin M., Rahman S.H., Vester-Christensen M.B., Behrens C., Kristensen C., Vakhrushev S.Y. (2015). Engineered CHO cells for production of diverse, homogeneous glycoproteins. Nat. Biotechnol..

[50] Pereira N.A., Chan K.F., Lin P.C., Song Z. (2018). The “less-is-more” in therapeutic antibodies: Afucosylated anti-cancer antibodies with enhanced antibody-dependent cellular cytotoxicity. MAbs.

[51] Krapp S., Mimura Y., Jefferis R., Huber R., Sondermann P. (2003). Structural analysis of human IgG-Fc glycoforms reveals a correlation between glycosylation and structural integrity. J. Mol. Biol..

[52] Shields R.L., Lai J., Keck R., O’Connell L.Y., Hong K., Meng Y.G., Weikert S.H.A., Presta L.G. (2002). Lack of fucose on human IgG1 N-linked oligosaccharide improves binding to human FcγRIII and antibody-dependent cellular toxicity. J. Biol. Chem..

[53] Hernandez I., Dhiman H., Klanert G., Jadhav V., Auer N., Hanscho M., Baumann M., Esteve-Codina A., Dabad M., Gómez J. (2019). Epigenetic regulation of gene expression in Chinese Hamster Ovary cells in response to the changing environment of a batch culture. Biotechnol. Bioeng..

[54] Yamane-Ohnuki N., Kinoshita S., Inoue-Urakubo M., Kusunoki M., Iida S., Nakano R., Wakitani M., Niwa R., Sakurada M., Uchida K. (2004). Establishment of FUT8 knockout Chinese hamster ovary cells: An ideal host cell line for producing completely defucosylated antibodies with enhanced antibody-dependent cellular cytotoxicity. Biotechnol. Bioeng..

[55] Malphettes L., Freyvert Y., Chang J., Liu P.Q., Chan E., Miller J.C., Zhou Z., Nguyen T., Tsai C., Snowden A.W. (2010). Highly efficient deletion of FUT8 in CHO cell lines using zinc-finger nucleases yields cells that produce completely nonfucosylated antibodies. Biotechnol. Bioeng..

[56] Imai-Nishiya H., Mori K., Inoue M., Wakitani M., Iida S., Shitara K., Satoh M. (2007). Double knockdown of alpha1,6-fucosyltransferase (FUT8) and GDP-mannose 4,6-dehydratase (GMD) in antibody-producing cells: A new strategy for generating fully non-fucosylated therapeutic antibodies with enhanced ADCC. BMC Biotechnol..

[57] Ronda C., Pedersen L.E., Hansen H.G., Kallehauge T.B., Betenbaugh M.J., Nielsen A.T., Kildegard H.F. (2014). Accelerating genome editing in CHO cells using CRISPR Cas9 and CRISPy, a web-based target finding tool. Biotechnol. Bioeng..

[58] Chan K.F., Shahreel W., Wan C., Teo G., Hayati N., Tay S.J., Tong W.H., Yang Y., Rudd P.M., Zhang P. (2016). Inactivation of GDP-fucose transporter gene (Slc35c1) in CHO cells by ZFNs, TALENs and CRISPR-Cas9 for production of fucose-free antibodies. Biotechnol. J..

[59] Joubert S., Guimond J., Perret S., Malenfant F., Elahi S.M., Marcil A., Parat M., Gilbert M., Lenferink A.E.G., Baardsnes J. (2022). Production of afucosylated antibodies in CHO cells by coexpression of an anti-FUT8 intrabody. Biotechnol. Bioeng..

[60] Varki A. (2008). Sialic acids in human health and disease. Trends Mol. Med..

[61] Bork K., Horstkorte R., Weidemann W. (2009). Increasing the sialylation of therapeutic glycoproteins: The potential of the sialic acid biosynthetic pathway. J. Pharm. Sci..

[62] Lin N., Mascarenhas J., Sealover N.R., George H.J., Brooks J., Kayser K.J., Gau B., Yasa I., Azadi P., Archer-Hartmann S. (2015). Chinese hamster ovary (CHO) host cell engineering to increase sialylation of recombinant therapeutic proteins by modulating sialyltransferase expression. Biotechnol. Prog..

[63] Anthony R.M., Nimmerjahn F., Ashline D.J., Reinhold V.N., Paulson J.C., Ravetch J.V. (2008). Recapitulation of IVIG anti-inflammatory activity with a recombinant IgG Fc. Science.

[64] Chung C.Y., Wang Q., Yang S., Yin B., Zhang H., Betenbaugh M. (2017). Integrated genome and protein editing swaps α-2,6 sialylation for α-2,3 sialic acid on recombinant antibodies from CHO. Biotechnol. J..

[65] Fischer S., Mathias S., Stadermann A., Yang S., Schmieder V., Zeh N., Schmidt N., Richter P., Wright S., Zimmermann E. (2022). Loss of a newly discovered microRNA in Chinese hamster ovary cells leads to upregulation of N-glycolylneuraminic acid sialylation on monoclonal antibodies. Biotechnol. Bioeng..

[66] Li F., Vijayasankaran N., Shen A., Kiss R., Amanullah A. (2010). Cell culture processes for monoclonal antibody production. MAbs.

[67] Varki A. (2007). Glycan-based Interactions Involving Vertebrate Sialic-acid-Recognizing Proteins. Nature.

[68] Amann T., Hansen A.H., Kol S., Hansen H.G., Arnsdorf J., Nallapareddy S., Voldborg B., Lee G.M., Andersen M.R., Kildegaard H.F. (2019). Glyco-engineered CHO cell lines producing alpha-1-antitrypsin and C1 esterase inhibitor with fully humanized N-glycosylation profiles. Metab. Eng..

[69] Clerc F., Reiding K.R., Jansen B.C., Kammeijer G.S.M., Bondt A., Wuhrer M. (2016). Human plasma protein N-glycosylation. Glycoconj. J..

[70] Koyuturk I., Kedia S., Robotham A., Star A., Brochu D., Sauvageau J., Kelly J., Gilbert M., Durocher Y. (2022). High-level production of wild-type and oxidation-resistant recombinant alpha-1-antitrypsin in glycoengineered CHO cells. Biotechnol. Bioeng..

[71] Amann T., Hansen A.H., Kol S., Lee G.M., Andersen M.R., Kildegaard H.F. (2018). CRISPR/Cas9-Multiplexed editing of Chinese hamster ovary B4Gal-T1, 2, 3, and 4 tailors N-glycan profiles of therapeutics and secreted host cell proteins. Biotechnol. J..

[72] Tian W., Ye Z., Wang S., Schulz M.A., van Coillie J., Sun L., Chen Y.H., Narimatsu Y., Hansen L., Kristensen C. (2019). The glycosylation design space for recombinant lysosomal replacement enzymes produced in CHO cells. Nat. Commun..

[73] Parenti G., Piganta C., Vajro P., Salerno M. (2013). New strategies for the treatment of lysosomal storage diseases. Int. J. Mol. Med..

[74] Platt F.M. (2018). Emptying the stores: Lysosomal diseases and therapeutic strategies. Nat. Rev. Drug Discov..

[75] Biel T.G., Faison T., Matthews A.M., Zou G., Ortega-Rodriguez U., Pegues M.A., Azer N., Gomez F., Johnson S., Rogstad S. (2022). An etanercept O-glycovariant with enhanced potency. Mol. Ther. Methods Clin. Dev..

[76] Mascarenhas J.X., Korokhov N., Burger L., Kassim A., Tuter J., Miller D., Borgschulte T., George H.J., Chang A., Pintel D.J. (2017). Genetic engineering of CHO cells for viral resistance to minute virus of mice. Biotechnol. Bioeng..

[77] Yang Z., Halim A., Narimatsu Y., Joshi H.J., Steentoft C., Schjoldager K.T.B.G., Schulz M.A., Sealover N.R., Kayser K.J., Bennett E.P. (2014). The GalNAc-type O-glycoproteome of CHO cells characterized by the SimpleCell strategy. Mol. Cell. Proteomics.

[78] Bratkovič T., Glavan G., Štrukelj B., Živin M., Rogelj B. (2012). Exploiting microRNAs for cell engineering and therapy. Biotechnol. Adv..

[79] Fischer S., Marquart K.F., Pieper L.A., Fieder J., Gamer M., Gorr I., Schulz P., Bradl H. (2017). miRNA engineering of CHO cells facilitates production of difficult-to-express proteins and increases success in cell line development. Biotechnol. Bioeng..

[80] Fischer S., Buck T., Wagner A., Ehrhart C., Giancaterino J., Mang S., Schad M., Mathias S., Aschrafi A., Handrick R. (2014). A functional high-content mirna screen identifies miR-30 family to boost recombinant protein production in CHO Cells. Biotechnol. J..

[81] Druz A., Son Y.J., Betenbaugh M., Shiloach J. (2013). Stable inhibition of mmu-miR-466h-5p improves apoptosis resistance and protein production in CHO cells. Metab. Eng..

[82] Sanchez N., Kelly P., Gallagher C., Lao N.T., Clarke C., Clynes M., Barron N. (2014). CHO Cell culture longevity and recombinant protein yield are enhanced by depletion of miR-7 activity via sponge decoy vectors. Biotechnol. J..

[83] Raab N., Mathias S., Alt K., Handrick R., Fischer S., Schmieder V., Jadhav V., Borth N., Otte K. (2019). CRISPR/Cas9-mediated knockout of microRNA-744 improves antibody titer of cho production cell lines. Biotechnol. J..

[84] Grilo A.L., Mantalaris A. (2019). Apoptosis: A mammalian cell bioprocessing perspective. Biotechnol. Adv..

[85] Cade C.E., Clark A.C., Bose K. (2015). Caspases-Key Players in Apoptosis. Proteases in Apoptosis: Pathways, Protocols and Translational Advances.

[86] Sung Y.H., Lee J.S., Park S.H., Koo J., Lee G.M. (2007). Influence of co-down-regulation of caspase-3 and caspase-7 by siRNAs on sodium butyrate-induced apoptotic cell death of Chinese hamster Ovary cells producing thrombopoietin. Metab. Eng..

[87] Safari F., Farajnia S., Behbahani A.B., Zarredar H., Barekati-Mowahed M., Dehghani H. (2020). Caspase-7 deficiency in Chinese hamster ovary cells reduces cell proliferation and viability. Biol. Res..

[88] Tang D., Lam C., Bauer N., Auslaender S., Snedecor B., Laird M.W., Misaghi S. (2022). Bax and Bak Knockout apoptosis-resistant Chinese hamster ovary cell lines significantly improve culture viability and titer in intensified fed-batch culture process. Biotechnol. Prog..

[89] MacDonald M.A., Barry C., Groves T., Martínez V.S., Gray P.P., Baker K., Shave E., Mahler S., Munro T., Marcellin E. (2022). Modeling apoptosis resistance in CHO cells with CRISPR-mediated knockouts of Bak1, Bax, and Bok. Biotechnol. Bioeng..

[90] Miao Z., Li Q., Zhao J., Wang P., Wang L., He H.P., Wang N., Zhou H., Zhang T., Lou X. (2018). Stable expression of infliximab in CRISPR/Cas9-mediated BAK1-deficient CHO cells. Biotechnol. Lett..

[91] Nguyen L.N., Baumann M., Dhiman H., Marx N., Schmieder V., Hussein M., Eisenhut P., Hernandez I., Koehn J., Borth N. (2019). Novel promoters derived from Chinese hamster ovary cells via in silico and in vitro analysis. Biotechnol. J..

[92] Dahodwala H., Lee K.H. (2019). The fickle CHO: A review of the causes, implications, and potential alleviation of the CHO cell line instability problem. Curr. Opin. Biotechnol..

[93] Yang Y., Chusainow J., Yap M.G. (2010). DNA methylation contributes to loss in productivity of monoclonal antibody-producing CHO cell lines. J. Biotechnol..

[94] Moritz B., Becker P.B., Göpfert U. (2015). CMV promoter mutants with a reduced propensity to productivity loss in CHO cells. Sci. Rep..

[95] Spahn P.N., Zhang X., Hu Q., Lu H., Hamaker N.K., Hefzi H., Li S., Kuo C., Huang Y., Lee J.C. (2022). Restoration of DNA repair mitigates genome instability and increases productivity of Chinese hamster ovary cells. Biotechnol. Bioeng..

[96] Pereira S., Kildegaard H.F., Andersen M.R. (2018). Impact of CHO metabolism on cell growth and protein production: An overview of toxic and inhibiting metabolites and nutrients. Biotechnol. J..

[97] Mulukutla B.C., Kale J., Kalomeris T., Jacobs M., Hiller G.W. (2017). Identification and control of novel growth inhibitors in fed-batch cultures of Chinese hamster ovary cells. Biotechnol. Bioeng..

[98] Young J.D. (2013). Metabolic flux rewiring in mammalian cell cultures. Curr. Opin. Biotechnol..

[99] Hetz C., Zhang K., Kaufman R.J. (2020). Mechanisms, regulation and functions of the unfolded protein response. Nat. Rev. Mol. Cell Biol..

[100] Kyeong M., Lee J.S. (2022). Endogenous BiP reporter system for simultaneous identification of ER stress and antibody production in Chinese hamster ovary cells. Metab. Eng..

[101] Lin P., Liu R., Alvin K., Wahyu S., Murgolo N., Ye J., Du Z., Song Z. (2021). Improving antibody production in stably transfected CHO cells by CRISPR-Cas9-mediated inactivation of genes identified in a large-scale screen with Chinese hamster-specific siRNAs. Biotechnol. J..

[102] Klanert G., Fernandez D.J., Weinguny M., Eisenhut P., Bühler E., Melcher M., Titus S.A., Diendorfer A.B., Gludovacz E., Jadhav V. (2019). A cross-species whole genome siRNA screen in suspension-cultured Chinese hamster ovary cells identifies novel engineering targets. Sci. Rep..

[103] Berting A., Farcet M.R., Kreil T.R. (2010). Virus susceptibility of Chinese hamster ovary (CHO) cells and detection of viral contaminations by adventitious agent testing. Biotechnol. Bioeng..

[104] Bethencourt V. (2009). Virus stalls Genzyme plant. Nat. Biotechnol..

[105] Nims R.W. (2006). Detection of adventitious viruses in biologicals—A rare occurrence. Dev. Biol..

[106] Rabenau H., Ohlinger V., Anderson J., Selb B., Cinatl J., Wolf W., Frost J., Mellor P., Doerr H.W. (1993). Contamination of genetically engineered CHO-cells by epizootic haemorrhagic disease virus (EHDV). Biologicals.

[107] Cotmore S.F., Tattersall P. (2007). Parvoviral host range and cell entry mechanisms. Adv. Virus Res..

[108] Merten O.W. (2002). Virus contaminations of cell cultures—A biotechnological view. Cytotechnology.

[109] (2011). ICH Guideline Q11 on Development and Manufacture of Drug Substances (Chemical Entities and Biotechnological/Biological Entities). ICH/425213/2011. https://www.ema.europa.eu/en/ich-q11-development-manufacture-drug-substances-chemical-entities-biotechnological-biological.

[110] Singh S.K. (2011). Impact of product-related factors on immunogenicity of biotherapeutics. J. Pharm. Sci..

[111] Gao S.X., Zhang Y., Stansberry-Perkins K., Buko A., Bai S., Nguyen V., Brader M.L. (2011). Fragmentation of a highly purified monoclonal antibody attributed to residual CHO cell protease activity. Biotechnol. Bioeng..

[112] Robert F., Bierau H., Rossi M., Agugiaro D., Soranzo T., Broly H., Mitchell-Loean C. (2009). Degradation of an Fc-fusion recombinant protein by host cell proteases: Identification of a CHO cathepsin D protease. Biotechnol. Bioeng..

[113] Jones M., Palackal N., Wang F., Gaza-Bulseco G., Hurkmans K., Zhao Y., Chitikila C., Clavier S., Liu S., Menesale E. (2021). “High-risk” host cell proteins (HCPs): A multi-company collaborative view. Biotechnol. Bioeng..

[114] Levy N.E., Valente K.N., Lee K.H., Lenhoff A.M. (2016). Host cell protein impurities in chromatographic polishing steps for monoclonal antibody purification. Biotechnol. Bioeng..

[115] Valente K.N., Lenhoff A.M., Lee K.H. (2015). Expression of difficult-to-remove host cell protein impurities during extended Chinese hamster ovary cell culture and their impact on continuous bioprocessing. Biotechnol. Bioeng..

[116] Fukuda N., Senga Y., Honda S. (2019). Anxa2 and Ctsd-knockout CHO cell lines to diminish the risk of contamination with host cell proteins. Biotechnol. Prog..

[117] Bailey-Kellogg C., Gutiérrez A.H., Moise L., Terry F., Martin W.D., de Groot A.S. (2014). CHOPPI: A web tool for the analysis of immunogenicity risk from host cell proteins in CHO-based protein production. Biotechnol. Bioeng..

[118] Dovgan T., Golghalyani V., Zurlo F., Hatton D., Lindo V., Turner R., Harris C., Cui T. (2021). Targeted CHO cell engineering approaches can reduce HCP-related enzymatic degradation and improve mAb product quality. Biotechnol. Bioeng..

[119] Li S.W., Yu B., Byrne G., Wright M., O’Rourke S., Mesa K., Berman P.W. (2019). Identification and CRISPR/Cas9 inactivation of the C1s protease responsible for proteolysis of recombinant proteins produced in CHO cells. Biotechnol. Bioeng..

[120] Li S.W., Wright M., Healey J.F., Hutchinson J.M., O’Rourke S., Mesa K.A., Lollar P., Berman P.W. (2020). Gene editing in CHO cells to prevent proteolysis and enhance glycosylation: Production of HIV envelope proteins as vaccine immunogens. PLoS ONE.

[121] Pugach P., Ozorowski G., Cupo A., Ringe R., Yasmeen A., de Val N., Derking R., Kim H.J., Korzun J., Golabek M. (2015). A native-like SOSIP.664 trimer based on an HIV-1 subtype B env gene. J. Virol..

[122] Byrne G., O’Rourke S.M., Alexander D.L., Yu B., Doran R.C., Wright M., Chen Q., Azadi P., Berman P.W. (2018). CRISPR/Cas9 gene editing for the creation of an MGAT1-deficient CHO cell line to control HIV-1 vaccine glycosylation. PLoS Biol..

[123] Kol S., Ley D., Wulff T., Decker M., Arnsdorf J., Schoffelen S., Hansen A.H., Jensen T.L., Gutierrez J.M., Chiang A.W.T. (2020). Multiplex secretome engineering enhances recombinant protein production and purity. Nat. Commun..

[124] Dick L.W., Qiu D., Mahon D., Adamo M., Cheng K.C. (2008). C-terminal lysine variants in fully human monoclonal antibodies: Investigation of test methods and possible causes. Biotechnol. Bioeng..

[125] Hu Z., Zhang H., Haley B., Macchi F., Yang F., Misaghi S., Elich J., Yang R., Tang Y., Joly J.C. (2016). Carboxypeptidase D is the only enzyme responsible for antibody C-terminal lysine cleavage in Chinese hamster ovary (CHO) cells. Biotechnol. Bioeng..

[126] Teixeira A.P., Stücheli P., Ausländer S., Ausländer D., Schönenberger P., Hürlemann S., Fussenegger M. (2022). CelloSelect—A synthetic cellobiose metabolic pathway for selection of stable transgenic CHO cell lines. Metab. Eng..

[127] Lai T., Yang Y., Ng S. (2013). Advances in mammalian cell cine development technologies for recombinant protein production. Pharmaceuticals.

[128] Zhang Q., Jiang B., Nelson L., Huhn S., Du Z., Chasin L.A. (2022). A multiauxotrophic CHO cell line for the rapid isolation of producers of diverse or high levels of recombinant proteins. Biotechnol. Prog..

[129] Hamaker N.K., Lee K.H. (2018). Site-specific integration ushers in a new era of precise CHO cell line engineering. Curr. Opin. Chem. Eng..

[130] Hilliard W., Lee K.H. (2021). Systematic identification of safe harbor regions in the CHO genome through a comprehensive epigenome analysis. Biotechnol. Bioeng..

[131] Gaidukov L., Wroblewska L., Teague B., Nelson T., Zhang X., Liu Y., Jagtap K., Mamo S., Tseng W.A., Lowe A. (2018). A multi-landing pad DNA integration platform for mammalian cell engineering. Nucleic Acids Res..

[132] Crawford Y., Zhou M., Hu Z., Joly J., Snedecor B., Shen A., Gao A. (2013). Fast identification of reliable hosts for targeted cell line development from a limited-genome screening using combined φC31 integrase and cre-lox technologies. Biotechnol. Prog..

[133] Sengupta S., George R.E. (2017). Super-enhancer-driven transcriptional dependencies in cancer. Trends Cancer.

[134] Lee Z., Raabe M., Hu W. (2021). Epigenomic features revealed by ATAC-seq impact transgene expression in CHO cells. Biotechnol. Bioeng..

[135] Inniss M.C., Bandara K., Jusiak B., Lu T.K., Weiss R., Wroblewska L., Zhang L. (2017). A novel Bxb1 integrase RMCE system for high fidelity site-specific integration of mAb expression cassette in CHO cells. Biotechnol. Bioeng..

[136] Chi X., Zheng Q., Jiang R., Chen-Tsai R.Y., Kong L.J. (2019). A system for site-specific integration of transgenes in mammalian cells. PLoS ONE.

[137] Sergeeva D., Lee G.M., Nielsen L.K., Grav L.M. (2020). Multicopy targeted integration for accelerated development of high-producing Chinese Hamster Ovary cells. ACS Synth. Biol..

[138] Lee J.S., Grav L.M., Pedersen L.E., Lee G.M., Kildegaard H.F. (2016). Accelerated homology-directed targeted integration of transgenes in Chinese Hamster Ovary cells via CRISPR/Cas9 and fluorescent enrichment. Biotechnol. Bioeng..

[139] Chavez A., Scheiman J., Vora S., Pruitt B.W., Tuttle M., Iyer E.P.R., Lin S., Kiani S., Guzman C.D., Wiegand D.J. (2015). Highly efficient Cas9-mediated transcriptional programming. Nat. Methods.

[140] Gilbert L.A., Larson M.H., Morsut L., Liu Z., Brar G.A., Torres S.E., Stern-Ginossar N., Brandman O., Whitehead E.H., Doudna J.A. (2013). CRISPR-mediated modular RNA-guided regulation of transcription in eukaryotes. Cell.

[141] Gilbert L.A., Horlbeck M.A., Adamson B., Villalta J.E., Chen Y., Whitehead E.H., Guimaraes C., Panning B., Ploegh H.L., Bassik M.C. (2014). Genome-scale CRISPR-mediated control of gene repression and activation. Cell.

[142] Dominguez A.A., Lim W.A., Qi L.S. (2016). Beyond Editing: Repurposing CRISPR-Cas9 for precision genome regulation and interrogation. Nat. Rev. Mol. Cell Biol..

[143] Yeo N.C., Chavez A., Lance-Byrne A., Chan Y., Menn D., Milanova D., Kuo C., Guo X., Sharma S., Tung A. (2018). An enhanced CRISPR repressor for targeted mammalian gene regulation. Nat. Methods.

[144] Pickar-Oliver A., Gersbach C.A. (2019). The next generation of CRISPR–Cas technologies and applications. Nat. Rev. Mol. Cell Biol..

[145] Ran F.A., Cong L., Yan W.X., Scott D.A., Gootenberg J.S., Kriz A.J., Zetsche B., Shalem O., Wu X., Makarova K.S. (2015). In vivo genome editing using *Staphylococcus aureus* Cas9. Nature.

[146] Wang F., Wang L., Zou X., Duan S., Li Z., Deng Z., Luo J., Lee S.Y., Chen S. (2019). Advances in CRISPR-Cas systems for RNA targeting, tracking and editing. Biotechnol. Adv..

[147] Konermann S., Lotfy P., Brideau N.J., Oki J., Shokhirev M.N., Hsu P.D. (2018). Transcriptome engineering with RNA-targeting type VI-D CRISPR effectors. Cell.

[148] Liu L., Pei D.S. (2022). Insights gained from RNA editing targeted by the CRISPR-Cas13 family. Int. J. Mol. Sci..

[149] Chen J.S., Doudna J.A. (2017). The chemistry of Cas9 and its CRISPR colleagues. Nat. Rev. Chem..

[150] Chavez A., Tuttle M., Pruitt B.W., Ewen-Campen B., Chari R., Ter-Ovanesyan D., Haque S.J., Cecchi R.J., Kowal E.J.K., Buchthal J. (2016). Comparison of Cas9 activators in multiple species. Nat. Methods.

[151] Shamie I., Duttke S.H., Karottki K.J.L.C., Han C.Z., Hansen A.H., Hefzi H., Xiong K., Li S., Roth S.J., Tao J. (2021). A Chinese hamster transcription start site atlas that enables targeted editing of CHO cells. NAR Genom. Bioinform..

[152] Karottki K.J.L.C., Hefzi H., Li S., Pedersen L.E., Spahn P.N., Joshi C., Duckerbauer D., Bort J.A.H., Thomas A., Lee J.S. (2021). A metabolic CRISPR-Cas9 screen in chinese hamster ovary cells identifies glutamine-sensitive genes. Metab. Eng..

[153] Doench J.G. (2017). Am I Ready for CRISPR? A user’s guide to genetic screens. Nat. Rev. Genet..

[154] Schmieder V., Novak N., Dhiman H., Nguyen L.N., Serafimova E., Klanert G., Baumann M., Kildegaard H.F., Borth N. (2021). A pooled CRISPR/AsCpf1 screen using paired grnas to induce genomic deletions in Chinese hamster ovary cells. Biotechnol. Rep..

[155] Xiong K., la Cour Karottki K.J., Hefzi H., Li S., Grav L.M., Li S., Spahn P., Lee J.S., Ventina I., Lee G.M. (2021). An optimized genome-wide, virus-free CRISPR screen for mammalian cells. Cell Rep. Methods.

[156] Canver M.C., Bauer D.E., Dass A., Yien Y.Y., Chung J., Masuda T., Maeda T., Paw B.H., Orkin S.H. (2014). Characterization of genomic deletion efficiency mediated by clustered regularly interspaced palindromic repeats (CRISPR)/Cas9 nuclease system in mammalian cells. J. Biol. Chem..

[157] Koonin E.V., Makarova K.S., Zhang F. (2017). Diversity, classification and evolution of CRISPR-Cas systems. Curr. Opin. Microbiol..

[158] Hille F., Charpentier E. (2016). CRISPR-Cas: Biology, mechanisms and relevance. Philos. Trans. R. Soc. Lond. B Biol. Sci..

[159] Zetsche B., Gootenberg J.S., Abudayyeh O.O., Slaymaker I.M., Makarova K.S., Essletzbichler P., Volz S.E., Joung J., van der Oost J., Regev A. (2015). Cpf1 is a single RNA-guided endonuclease of a class 2 CRISPR-Cas system. Cell.

[160] Schweickert P.G., Wang N., Sandefur S.L., Lloyd M.E., Konieczny S.F., Frye C.C., Cheng Z. (2021). CRISPR/Cas12a-mediated CHO genome engineering can be effectively integrated at multiple stages of the cell line generation process for bioproduction. Biotechnol. J..

[161] Bydlinski N., Coats M.T., Maresch D., Strasser R., Borth N. (2020). Transfection of glycoprotein encoding mRNA for swift evaluation of N-glycan engineering strategies. Biotechnol. Prog..

[162] Zhang X.H., Tee L.Y., Wang X.G., Huang Q.S., Yang S.H. (2015). Off-target effects in CRISPR/Cas9-mediated genome engineering. Mol. Ther. Nucleic Acids.

[163] Lee N., Shin J., Park J.H., Lee G.M., Cho S., Cho B.K. (2016). Targeted gene deletion using DNA-free RNA-guided Cas9 nuclease accelerates adaptation of CHO cells to suspension culture. ACS Synth. Biol..

[164] Ramakrishna S., Dad A.B.K., Beloor J., Gopalappa R., Lee S.K., Kim H. (2014). Gene disruption by cell-penetrating peptide-mediated delivery of Cas9 protein and guide RNA. Genome Res..

[165] Rees H.A., Liu D.R. (2018). Base editing: Precision chemistry on the genome and transcriptome of living cells. Nat. Rev. Genet..

[166] Rojek J.B., Basavaraju Y., Nallapareddy S., Dubhe D., Ocaña B.B., Baumgartner R., Grabenhorst E., Tharmalingam T., Münch R., Rhiel L. (2023). Expanding the CRISPR toolbox for Chinese hamster ovary cells with comprehensive tools for Mad7 genome editing. Biotechnol. Bioeng..

[167] Zetsche B., Heidenreich M., Mohanraju P., Fedorova I., Kneppers J., DeGennaro E.M., Winblad N., Choudhury S.R., Abudayyeh O.O., Gootenberg J.S. (2017). Multiplex gene editing by CRISPR–Cpf1 using a single crRNA array. Nat. Biotechnol..

[168] Gao Y., Xiong X., Wong S., Charles E.J., Lim W.A., Qi L.S. (2016). Complex transcriptional modulation with orthogonal and inducible dCas9 regulators. Nat. Methods..

[169] Rupp O., MacDonald M.L., Li S., Dhiman H., Polson S., Griep S., Heffner K., Hernandez I., Brinkrolf K., Jadhav V. (2018). A reference genome of the Chinese hamster based on a hybrid assembly strategy. Biotechnol. Bioeng..

[170] Hilliard W., MacDonald M.L., Lee K.H. (2020). Chromosome-scale scaffolds for the Chinese hamster reference genome assembly to facilitate the study of the CHO epigenome. Biotechnol. Bioeng..

[171] Kleinstiver B.P., Pattanayak V., Prew M.S., Tsai S.Q., Nguyen N.T., Zheng Z., Joung J.K. (2016). High-fidelity CRISPR–Cas9 nucleases with no detectable genome-wide off-target effects. Nature.

[172] Nishimasu H., Shi X., Ishiguro S., Gao L., Hirano S., Okazaki S., Noda T., Abdudayyeh O.O., Gootenberg J.S., Mori H. (2018). Engineered CRISPR-Cas9 nuclease with expanded targeting space. Science.

[173] Hu J.H., Miller S.M., Geurts M.H., Tang W., Chen L., Sun N., Zeina C.M., Gao X., Rees H.A., Lin Z. (2018). Evolved Cas9 variants with broad PAM compatibility and high DNA specificity. Nature.

[174] Chen W.C.W., Gaidukov L., Lai Y., Wu M.R., Cao J., Gutbrod M.J., Choi G.C.G., Utomo R.P., Chen Y.C., Wroblewska L. (2022). A synthetic transcription platform for programmable gene expression in mammalian cells. Nat. Commun..

[175] Carver J., Kern M., Ko P., Greenwood-Goodwin M., Yu X.C., Duan D., Tang D., Misaghi S., Auslaender S., Haley B. (2021). A ribonucleoprotein-based decaplex CRISPR/Cas9 knockout strategy for CHO host engineering. Biotechnol. Bioeng..

